# One-pot synthesis of triazines as potential agents affecting cell differentiation

**DOI:** 10.1007/s00706-018-2212-0

**Published:** 2018-05-15

**Authors:** Thomas Linder, Michael Schnürch, Marko D. Mihovilovic

**Affiliations:** Institute of Applied Synthetic Chemistry, TU Wien, Getreidemarkt 9/163, 1060 Vienna, Austria

**Keywords:** Cell differentiation, Triazine, One-pot synthesis, Nucleophilic substitution

## Abstract

**Abstract:**

This paper outlines the synthesis of a number of structural analogs of 3-[(4,6-diphenoxy-1,3,5-triazin-2-yl)amino]benzoic acid which represent compounds with potential cardiogenetic activity. A one-pot protocol was developed for swift functionalization of the 1,3,5-triazine core without the need of isolating intermediates. The developed route starts from readily available 2,4,6-trichloro-1,3,5-triazine, displacing the chlorine atoms sequentially by aryloxy, arylamino, or arylthio moieties to enable access to molecules with three different substituents of this type in good yields. To facilitate purification, *tert*-butyl, methyl, and ethyl ester derivatives of the target compounds were initially synthesized. The *tert*-butyl esters could be readily hydrolyzed to the desired compounds, while reduction of the methyl and ethyl esters gave the corresponding benzylic alcohols in high yields, thereby expanding the substrate scope for future relevant cell assays.

**Graphical abstract:**

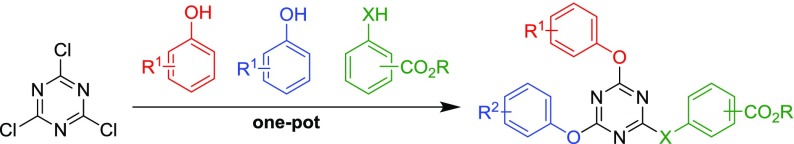

## Introduction

During the development of animals and humans from a zygote to a multicellular biological system, the organism needs to grow by cell fission and dedicate newly formed cells to particular purposes. The process that converts those cells of the early stages of life (the embryonic stem cells) to specialized tissue is known as cell differentiation. Apart from a merely biological interest, the subject is of great importance to the medical sciences, as it promises to hold the key for replacing damaged or lost tissue and organs [1–5].

Cardiovascular diseases are among the most important causes of death globally [6] and an estimated average number of 15 years of life are lost because of a myocardial infarction [7]. As about 34% of the patients experiencing a coronary attack will die due to this event, these figures point out the importance of new methods for cardiac repair. Apart from replacement by tissue transplant, the restoration by activation of resident (cardiac) stem cells or regeneration by the formation of cardiomyocytes from progenitor or stem cells has been given much attention [8–10]. This is particularly important in the case of renewing dead cardiomyoblasts in the wage of a condition such as myocardial infarction, because the mammalian (i.e., human) heart responds to tissue damage by scarring rather than regeneration [11]. Human embryonic stem cells have shown the potential to develop into cardiomyocytes in vitro. One approach is gene therapy, which involves the use of viral vectors for genetic manipulation and makes it difficult to achieve approval by health regulating bodies for clinical use [12, 13]. Similarly, the use of viruses to obtain induced pluripotent stem (iPS) cells from differentiated cells for reprogramming also raises safety issues, while the efficiency of plasmids for this purpose is extremely low [14]. The use of small organic molecules to trigger cell differentiation represents an alternative, which could be carried out ex vivo and the so-formed cardiomyocytes subsequently implanted.

**Fig. 1:**
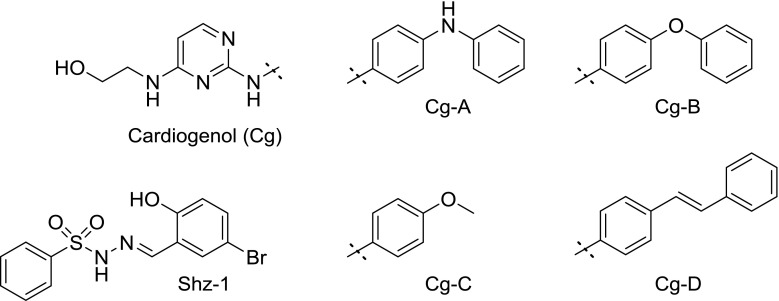
Cardiogenols and Shz-1 as literature-known compounds with cardiomyogenic activity

**Fig. 2:**
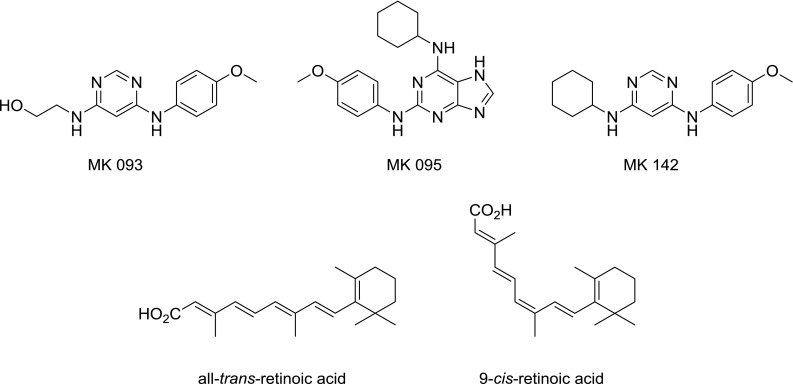
Examples of compounds with cardiomyogenic activity. Top: synthesized in our group. MK 142 could be shown to produce beating heart muscle cells from W4 embryonic stem cells. Bottom: Retinoic acid isomers

In 2004, a screen with mouse embryonic carcinoma P19 cells showed that certain 2-pyrimidinamines, termed cardiogenols A–D (Fig. 1) up-regulated the expression of an artificial gene containing the promoter for the atrial natriuretic factor (ANF) [15]. This is a polypeptide hormone, synthesized and excreted primarily by cardiomyocytes, and therefore considered a cardiac-specific marker protein [16, 17]. Considerable research has been conducted in our group to substantiate the cardiogenetic potential of these molecules, as well as finding new ones with promising results in the relevant biological screens (as exemplified in Fig. 2 top) [18–20].

Retinoic acid, which is an active metabolite of retinol (vitamin A) and occurs naturally in the all-*trans* (atRA) and 9-*cis* form (Fig. 2 bottom), has been shown to take part in a multitude of physiological functions, notably during the development of the organism and the proliferation of cells [21].

Among these are the inhibition of the serotonin-activated proliferation of aortic smooth muscle cells in primary culture (as shown in dogs) and inhibition of cell proliferation in cultivated chick embryonic vascular smooth muscle cells [22, 23], to the extent that retinoids, such as atRA, have been used clinically for anti-cancer treatments and therapies in dermatology [24]. On the other hand, there have been indications that retinoic acid may enhance, rather than suppress, proliferation of smooth muscle cells [25]. Examples like these illustrate that the physiological effects of retinoic acid are very complex and highly dependent on the concentration at which it is applied, as well as the developmental stage of the cell at the time of exposure.

More important to this work, however, is the finding that retinoids generally inhibit cell proliferation and promote cardiac differentiation of stem cells [26]. While applying small molecules for heart muscle formation is a promising approach, applying retinoic acid itself to stem cells has multiple issues. Given its polyolefinic structure, it is rather reactive in the presence of oxygen and light [27]. It is also isomerized under physiological conditions and enters the metabolism of the cell [28]. As indicated above, this natural compound also fulfills many other roles in cell biochemistry and responses of the cell exposed to it depend crucially on the concentration and the state of cell, because its interaction is unlikely to be limited to one target only [29, 30]. This aggravates screening and makes the availability of synthetic retinoids highly desirable.

**Fig. 3:**
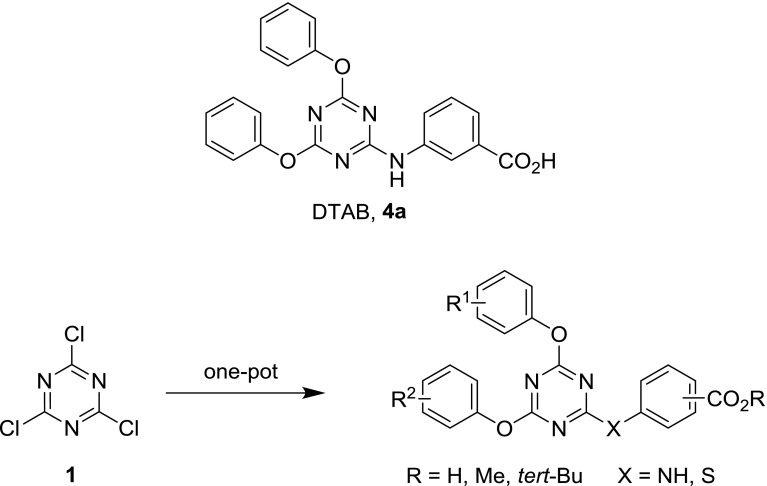
Parent literature compound and the envisioned synthetic route

Recently, it was determined that the heterocyclic compound 3-[(4,6-diphenoxy-1,3,5-triazin-2-yl)amino]benzoic acid (DTAB, compound **4a**, Fig. 3) is an RARβ and RARγ selective ligand, activating retinoic acid signaling [31]. This compound served as a starting point for the present work, in which a method was developed which enables the preparation of compounds with this scaffold from 2,4,6-trichloro-1,3,5-triazine in a sequential, one-pot fashion (Fig. 3), making use of the step-wise reactivity of the chloride substitution process.

Although not strictly analogous to the pyrimidine compounds shown in Figs. 1 and 2, this addition of a nitrogen atom to the core ring structure (by shifting from the pyrimidine to the 1,3,5-triazine heterocycle) extends the scaffold range in a logical bioisosteric modification and complements the previous studies already conducted in the area of cardiogenesis in our group.

## Results and discussion

Symmetrically trisubstituted triazines can be prepared by protic or Lewis acid- or base-catalyzed cyclotrimerization of nitriles or acid-catalyzed cyclocondensation of imidic esters (Scheme 1) [32].

**Figure:**
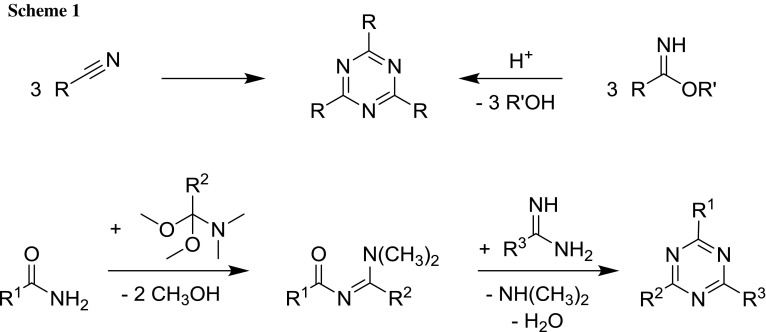

1,3,5-Triazines with three different substituents are accessible by condensation of acylamidines (themselves prepared from amides and amide acetals) with other amidines (Scheme 1) [28].

2,4,6-Trichloro-1,3,5-triazine (cyanuric chloride, **1**) is an electron-poor heterocycle and behaves as a heterocyclic acid chloride analog, with its reactive chlorine atoms being easily substituted in S_N_Ar reactions. Therefore, it can serve as starting material for (differently) substituted 1,3,5-trazines, where the substituents are linked via nucleophilic heteroatoms, such as oxygen, nitrogen, and sulfur.

To gain experience with nucleophilic aromatic substitution on the 1,3,5-triazine scaffold, a known procedure to produce 2-phenoxy-4,6-dichloro-1,3,5-triazine (**2a**) was repeated [33]. In this procedure, 2,4,6-trichloro-1,3,5-triazine was combined with one equivalent of phenol and *N*,*N*-diisopropylethylamine (DIPEA) in THF at 0 °C (Scheme 2 top). While the authors state that no purification was required (short of removing the amine hydrochloride by filtration) to afford **2a** in 92% yield, GC–MS analysis revealed that about 10% of the disubstituted triazine **3a** was formed, as well. Although this could be removed by re-crystallization, such a byproduct ratio would have a negative impact on the one-pot procedure in which three sequential nucleophilic replacements were aimed at without any work-up of intermediates. Thus, on repetition of this synthesis, the reaction temperature was lowered to − 15 °C initially and the reaction time was extended to allow for complete conversion, which avoided formation of byproduct and gave **2a** in 95% without further purification (Scheme 2 top).

**Figure:**
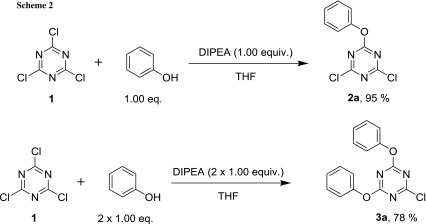

This reaction proceeds swiftly in the beginning, with the appearance of the hydrochloride salt after a few minutes. Since combining the two components under basic conditions is noticeably exothermic, it was decided to add the phenolic compounds as THF solutions to ensure the reproducibility of byproduct suppression. Disubstituted **3a** was prepared in this way, adding two equivalents in succession. After the amount of the mono-substituted intermediate **2a** had decreased to less than 2% (GC–MS), the reaction mixture was worked up and gave **3a** in 78% after purification (Scheme 2 bottom).

In general, neither of these two intermediates is very stable; they decompose visibly if stored at room temperature for a period of a few months. Unlike the actual target trisubstituted triazines, the disubstituted compounds are also attacked by DMSO, which precludes direct NMR shift comparison in the same solvent, because the latter are generally not soluble enough in, e.g., CDCl_3_ (trichlorotriazine itself reacts vigorously with DMSO, by attack of the oxygen) [34]. This actually speaks in favor of a method which omits isolation and purification of such reactive intermediates and makes a one-pot protocol all the more appealing.

An unexpected behavior occurred when two different aryloxy residues were attempted to be incorporated into the triazine scaffold. In the first step, 4-chlorophenol (an electron-deficient phenol) was reacted with starting material **1,** and the reaction was checked for completion by TLC and GC–MS. Then, with addition of phenol in the second step, three products emerged (Scheme 3): in addition to expected **3b**, compounds **3c** and **3a** were also detected, in the approximate ratio of 8:2:1. A very similar product distribution was obtained when 2- or 3-chlorophenol was used in step one. By contrast, starting from purified **2a** and reacting it with 4-chlorophenol under the same conditions, almost none of the undesired products were formed (ratio of 97:2:1).

**Figure:**
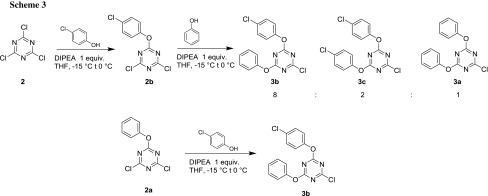

These findings can be readily explained by looking at the electronic features of the applied phenols. Owing to the electron-withdrawing substituent, chlorophenol(s) can also serve as a leaving group and remain present in the reaction mixture after replacement by phenol. In this displacement reaction, **2a** is formed as an intermediate. Both **2b** and **2a** can then form substitution products with either phenol (added to the mixture for the second step) or chlorophenol (liberated in the displacement) and, hence, lead to the observed product distribution. This has two implications: if both electron-rich(er) and electron-poor(er) phenoxy substituents have to be incorporated at the triazine core, then the order of addition of the two phenols is important: the electron-richer(er) has to be added prior to the electron-poor(er). Knowing this, the final step of the sequence could be investigated.

A preliminary experiment showed that 3-aminobenzoic acid reacts with **3a** under much the same conditions (DIPEA as the base) as in the preparation of **2a** and **3a** to give the parent literature compound **4a** (DTAB, Scheme 4).

**Figure:**
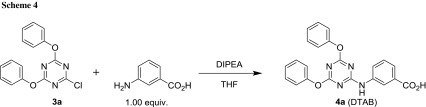

As a somewhat troubling property, it was found that none of these trisubstituted triazine compounds (with or without the presence of a carboxylic acid function) could be detected in either GC or GC–MS analysis, limiting reaction control to TLC analysis. In general, the triazinylaminobenzoic acids were crystalline materials, sparingly-to-moderately soluble in common organic solvents (e.g., very low solubility in CHCl_3_, MeOH, Et_2_O, MeCN; best solubility was found with 1,4-dioxane). It, therefore, appeared promising to prepare a variety of these compounds in one pot by starting from trichlorotriazine, incorporating first two different phenoxy substituents and subsequently adding the amine compound.

While the reaction itself worked as expected, a severe purification problem occurred: as chromatographic separation of carboxylic acids was not feasible, re-crystallization remained the only means of purification. Because the step-wise reactivity of trichlorotriazine in the nucleophilic substitution was neither fully selective, nor complete, unwanted other triazinyl benzoic acids, mostly those with two identical aryloxy substituents, were also obtained. These persisted in the 5–10% range as judged by HPLC analysis after re-crystallization from various solvents (CHCl_3_/*n*-hexane, toluene, and EtOH). Therefore, target compounds could not be purified to a satisfactory degree.

In light of these purification issues, carboxylic acids were masked as their methyl (and, in one case, ethyl) esters, i.e., retaining the one-pot protocol but using an alkyl aminobenzoate in the third substitution step (Scheme 5). This opened the possibility for chromatographic separation.

**Figure:**
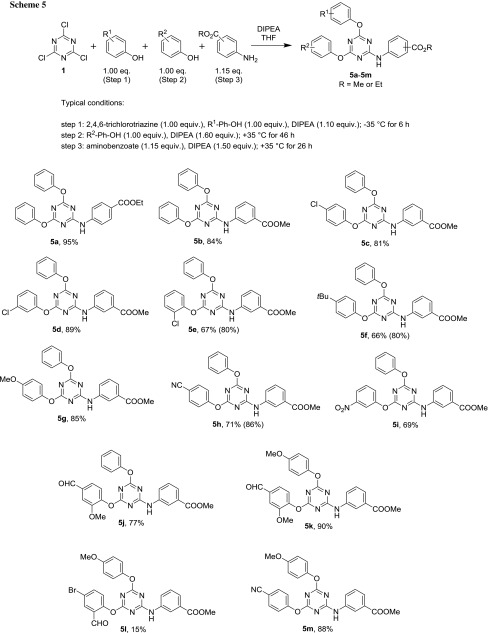

To reduce disubstitution in the first nucleophilic displacement even further, the solutions containing the phenols and the base were also cooled to the reaction temperature (− 35 °C) prior to dropwise addition (at the reported temperature of 0 °C [20, 35], the reaction is less selective, as stated above). In general, care needs to be taken as to drive the reaction as far to completion as possible, however, without losing selectivity. This is why the reaction times tended to be very long (up to 47 h), while the reaction temperature was not taken beyond + 40 °C. The results are summarized in Scheme 5.

The reactions, which were carried out using readily available phenols proceeded very well in most cases. Various electron-rich and -poor phenols were used to investigate the scope of this method, also beyond common pharmacophores such as chloro and methoxy substituents. As for the performance of the reaction, there is no significant difference in yields of compounds bearing electron donating *p*-*tert*-butyl and *p*-methoxy, versus electron-withdrawing *p*-, *m*-, *o*-chloro, *p*-cyano, and *m*-nitro substituents. One exception is compound **5l**: the second nucleophilic substitution with 5-bromo-2-hydroxybenzaldehyde was not complete, even after 46 h, and considerable amount of the starting material was recovered by column chromatography. In addition, purification was plagued with separating this compound from byproducts as it showed great tailing on the column. Therefore, after two chromatographic runs, only 15% could be obtained. By contrast, more electron-rich aldehydes, such as vanillin (leading to **5j** and **5k**), worked quite well and difficulties with the use of the 3-aminobenzoate in the third step did not arise, probably owing to the low temperature conditions.

Compounds **5e**, **5f**, and **5h** were first purified by re-crystallization. As this did not eliminate byproducts completely (i.e., differently substituted triazines; impurities in the 1–5% range), subsequent column chromatography was applied for final purification, but the overall decrease in yield was significantly larger than the amount of impurity initially present (Scheme 5, yields before column are in parentheses).

To evaluate the importance of a carboxylic functional group on the aniline moiety in positions 3 and 4 for biological activity, two more compounds were prepared devoid of this functionality. Since the two phenoxy substituents were identical, two equivalents of phenol were used in one go rather than adding them sequentially as in the cases above (Scheme 6). Being a good nucleophile, thiophenol was also incorporated well into the triazine ring.

**Figure:**
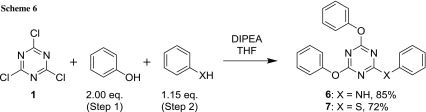

While **6** is a known compound [36, 37], a literature reference to **7** could not be found. It offers the possibility of another linking group besides NH and S, by oxidation of the sulfide to the sulfoxide (SO) or the sulfone (SO_2_). This could be used if one wishes to reverse the role of this part of the molecule in hydrogen bonding, switching it from a hydrogen donor to an acceptor.

Next, the obtained benzoic ester products had to be hydrolyzed to the corresponding carboxylic acids. Since methyl and ethyl esters are usually best cleaved by irreversible base hydrolysis, this was attempted first. However, in all the attempts and seemingly irrespective of temperature (− 20 to + 50 °C), the hydroxide ion attacked the electron-deficient triazine carbon bonded to the phenoxy substituents and released the phenol, preferentially the more electron-deficient one. This process also consumed the amount of base available, leaving unhydrolyzed ester behind. In addition, a variation of hydroxide-promoted hydrolysis in a non-aqueous environment did not bring the desired results [38].

On the other hand, the model compounds proved inert towards acid hydrolysis, short of exceptionally harsh conditions leading to its destruction. Since all attempts to cleave the methyl esters proved to be futile, the use of alternative esters was considered.

The *tert*-butyl ester group is well known to be readily cleaved by acid, because the stable *tert*-butyl carbenium ion can act as a leaving group on protonation of the adjacent oxygen atom [39]. Moreover, no water is required in this process, the only byproduct is gaseous isobutene (formed by elimination from the carbenium ion) and this deprotection can be carried out under mild conditions. For this reason, 3-*tert*-butylaminobenzoate was used in a one-pot sequence analogously to the synthesis of the methyl esters (Scheme 7).

**Figure:**
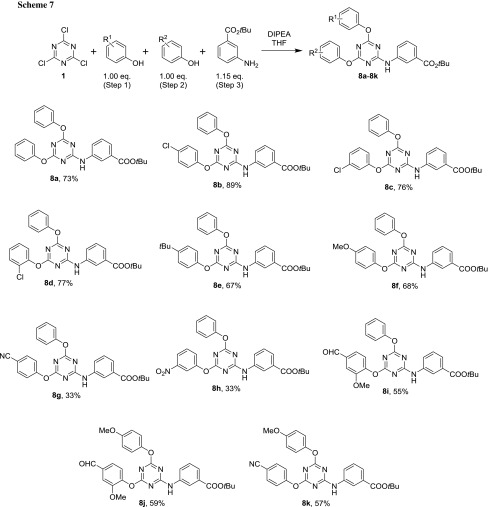

In an overall view, this reaction produced somewhat lower yields and appeared to be slower (Scheme 7) than the synthesis of the corresponding methyl esters. The *tert*-butyl group was stable on silica during chromatography. Work-up had to include an aqueous extraction step with diluted hydrochloric acid to remove the 3-*tert*-butylaminobenzoate, which was used in slight excess (1.15 equivalents), because this material had very similar *R*_*f*_ values to products **8a**–**8j**. This operation was carried out quickly to avoid ester cleavage prior to chromatographic separation—even though the corresponding carboxylic acid was eventually desired, because only the ester could be purified chromatographically. The solution containing the product was then treated with NaHCO_3_ solution (for neutralization) and brine before evaporation and column chromatography. The compounds also turned out to be generally more soluble and less inclined to crystallize than the corresponding methyl esters because of the non-polar *tert*-butyl group, although crystalline materials could be obtained in all cases.

As indicated above, the *tert*-butyl group can be removed easily under moderately strong acidic conditions. The classic removal condition involves the use of trifluoroacetic acid [40] (p*K*_*A*_ = 0.23) [41], but formic acid [42] (p*K*_*A*_ = 3.77) [43] can also be used. This is, therefore, a milder method, which is compatible with a large number of functional groups. First, formic acid, dissolved in CH_2_Cl_2_ was tried at room temperature, but this did not advance the reaction. However, upon heating to 40 °C, the reaction proceeded as observed by TLC after several hours. Finally, it proved a very simple and effective operation to take up the compound solely in formic acid (with some sonication to aid dissolution) and stir the solution at slightly elevated temperatures (40–50 °C). A large excess of formic acid (4 cm^3^ for, e.g., 0.1 mmol) was used to make sure that the intermediately formed and very strongly electrophilic *tert*-butyl cation would not be likely to react with either the starting material or the product (as is sometimes the case) [44] in, e.g., an electrophilic aromatic substitution reaction of the carbocycles, before it is eliminated to isobutene. In all but one case (compound **4j**), the cleavage reaction was complete within 1 h.

Purification was not required; work-up was limited to adding diethyl ether after concentrating the reaction product to dryness and to remove the solvent again in vacuo to aid evaporation of the formic acid. Thus, the desired carboxylic acids **4a**–**4k** were obtained in quantitative yields (Scheme 8).

**Figure:**
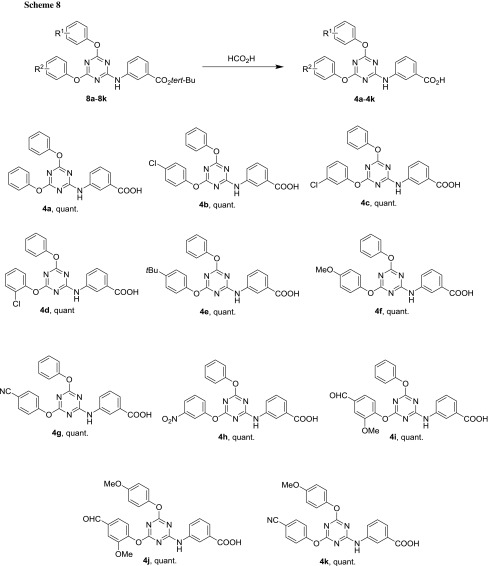

From the viewpoint of biological activity, it is interesting to compare the changes that occur when the carboxylic acid function—common to all structures so far, except **6** and **7**—is replaced by another group. The hydroxyl functional group of a benzylic alcohol represents such a change; it is also polar and capable of hydrogen bonding. These compounds were obtained by reducing the corresponding methyl and ethyl esters, since the *tert*-butyl group of compounds in Scheme 7 would be quite resistant to hydride attack. Usually, such a transformation is accomplished by a highly reactive complex metal hydride, notably lithium aluminium hydride. Since it was already obvious that strong nucleophiles are not tolerated very well due to the reactivity of the C–O bond of the substituted triazines, a less aggressive method would be highly sought-after. Based on the higher electronegativity of boron (2.0 of B vs. 1.6 of Al), the frequently used sodium borohydride is a less reactive reducing agent but usually falls short of transforming esters accordingly. However, additional reactivity can be gained by changing the counter-ion to lithium, which offered the prospect of having just the right reductive power to accomplish the task. Literature suggested that the reactivity of the borohydride has been enhanced by the addition of lithium chloride [45], and fast reductions with this reagent have been performed by microwave irradiation [46]. Moreover, it has been reported that reducible groups, such as NO_2_ and CN, can be preserved under lithium borohydride conditions [47].

To test the reduction, compounds **5c**–**5e** were used again (Scheme 9). An initial attempt to carry out such a reduction of **5e** using 1.1 equivalents of NaBH_4_ in the presence of 2.0 equivalents of LiCl by the conventional heating at 65 °C in anhydrous THF did not bring about this transformation after 18 h. Higher temperature (75 °C, and then 90 °C) only led to compound decomposition as indicated by multiple spots on TLC. Since also other related conditions did not lead to the desired reduction in acceptable amounts, another reducing agent had to be considered. Based on the idea that increased steric bulk may prevent the hydride ion from attacking the electrophilic triazine carbons, diisobutylaluminium hydride was tried under standard conditions in anhydrous CH_2_Cl_2_ under argon at − 70 °C. Although a large excess of this reagent (up to 5.1 equivalents) had to be used to complete the reaction relatively quickly (around 1–3 h), the desired conversion could be completed with good yields (Scheme 10). Most importantly, no phenols were detected, and purification was only needed to remove some residual starting material that had escaped TLC analysis (due to its low concentration) during the reaction. However, triazinyl benzoic esters bearing nitro and cyano groups were not attempted, because these functions are both readily reduced, as well.

**Figure:**
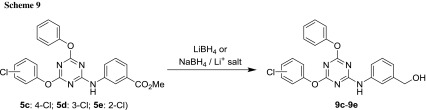

**Figure:**
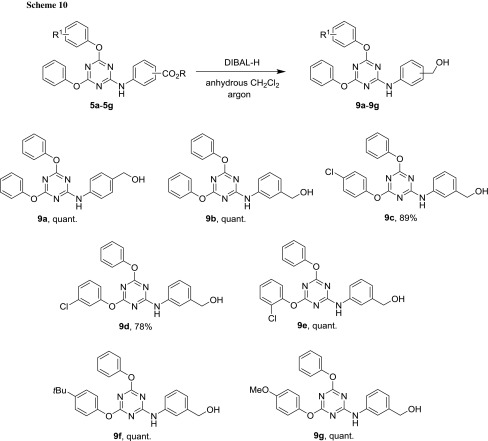

## Conclusion

In this work, it was demonstrated that derivates of the triazine compound DTAB, **4a**, bearing three different substituents, can be prepared in a one-pot protocol. However, if carboxylic acids akin to DTAB are desired, separation problems emerge; and it is, therefore, necessary to mask the carboxylic function to facilitate purification. The compounds in question are still labile toward nucleophiles (hydroxide, hydride, and methoxide), which prevents smooth basic hydrolysis of methyl esters and their reduction to the corresponding benzylic alcohols with lithium or sodium borohydride. Still, they can be converted using the more sterically demanding diisobutylaluminium hydride in good yields. In addition, the triazine scaffold is inert toward acid, which allows the facile and operationally simple acid-promoted cleavage of *tert*-butyl esters of the corresponding carboxylic acids, affording the latter quantitatively. Biological results have been recently disclosed in a patent application [48, 49].

## Experimental

Chemicals: Unless otherwise noted, chemicals were purchased from commercial suppliers and used without further purification. DIBAL-H solution in *n*-hexane was reaction-titrated according to a literature procedure (3×) and the content of active DIBAL-H was determined by standard ^1^H NMR spectroscopy [50]. Dry solvents were obtained by passing pre-dried material through a cartridge containing activated alumina (solvent dispensing system) and stored under N_2_. Microwave reactions were performed using a Biotage Initiator 2.5 laboratory microwave device. Chromatography: Flash column chromatography was performed on Merck silica gel 60 (40–63 μm). Separations were carried out either using a Büchi Sepacore system (MPLC) or by hand column (as noted). For thin-layer chromatography (TLC), aluminium backed Merck silica gel 60 with fluorescence indicator F_254_ was used. For preparative TLC, Analtech Uniplate silica gel GF (20 × 20 cm, 1000 µm) glass-backed plates with fluorescence indicator UV254 were used. Distillation: *Kugelrohr* distillation was carried out using a Büchi GKR-51 apparatus. Melting points were determined using a Kofler-type Leica Galen III micro hot stage microscope or a Stanford Research Systems MPA100 OptiMelt Automatic Melting Point System. Data are given in 0.5 °C intervals. GC–MS: GC–MS runs were performed on a Thermo Finnigan Focus GC/DSQ II using a standard capillary column BGB 5 (30 m × 0.32 mm ID) and applying the following standardized temperature profile: 2 min at 100 °C/ramp 18 °C min^−1^ until 280 °C/5 min at 280 °C. Electron ionization was used (70 eV); all fragment signals (*m*/*z*) at/over mass 100 and at/over 10% relative intensity are indicated. NMR spectroscopy: NMR spectra were recorded from DMSO-*d*_*6*_ or CDCl_3_ solutions on a Bruker AC 200 (200 MHz) or a Bruker Avance UltraShield (400 MHz) spectrometer (as indicated), and chemical shifts are reported in ppm relative to the nominal residual solvent signals [51]: CDCl_3_: *δ* = 7.26 ppm (^1^H), *δ* = 77.16 ppm (^13^C); DMSO-*d*_*6*_: *δ* = 2.50 (^1^H), *δ* = 39.52 (^13^C). DEPT-135 or *J*-modulation pulse sequences (APT) were used to aid in the multiplet assignment in the ^13^C spectra.

**Fig. 4:**
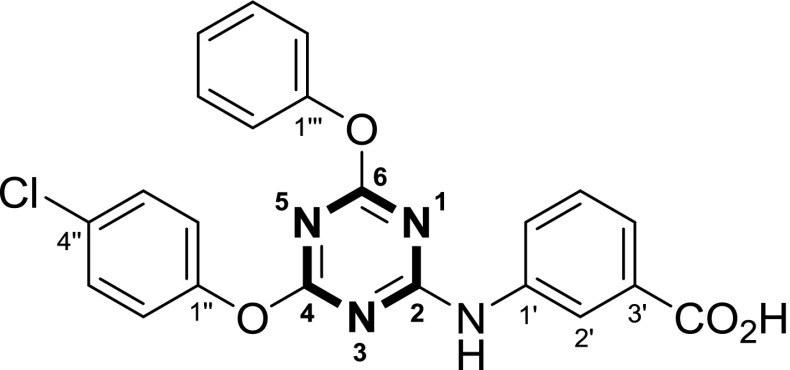
Explanation of code for peak assignment

Numbering of the atoms in the synthesized products parallels the priority of the individual cycles of the molecules, as inferred from systematic nomenclature. Therefore, the hydrogen and carbon positions belonging to the ring with the highest priority are referred to as H1, H2, H3,… and C1, C2, C3,…, respectively. The numbers designate the order of the atoms within a particular ring system. The atoms in the second ring are indicated with one prime (H1’, H2’, H3’,… and C1’, C2’, C3’,…), in the next ring with two primes, etc. However, the core 1,3,5-triazine cycle is given highest priority in all cases. This is done regardless that a compound may actually bear its name-giving functional group on one of the lower priority rings, as the suffix …*acid* implies for the example, as shown in Fig. 4.

In addition, all compounds are drawn in such a way that they follow the ’, ”, ’” order of the second, third, and fourth ring in the clockwise manner as shown. Identified signals were marked with an asterisk (*) when assignment was equivocal.

### NMR characteristics of 2,4,6-trisubstitued 1,3,5-triazines

**Fig. 5:**
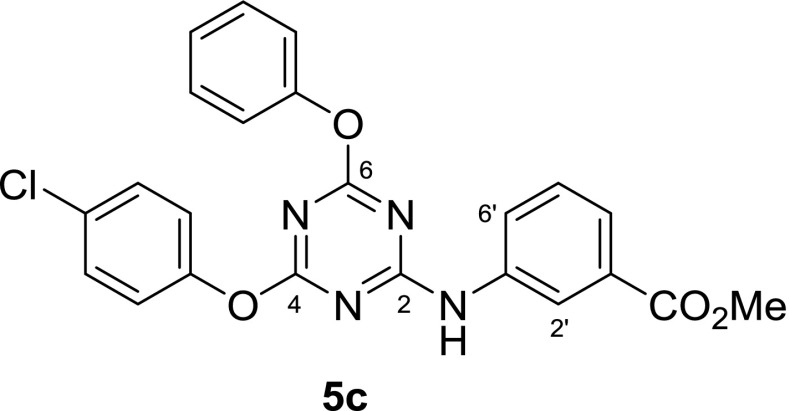
Compound **5c**, an example for unexpected ^13^C NMR behavior

**Fig. 6:**
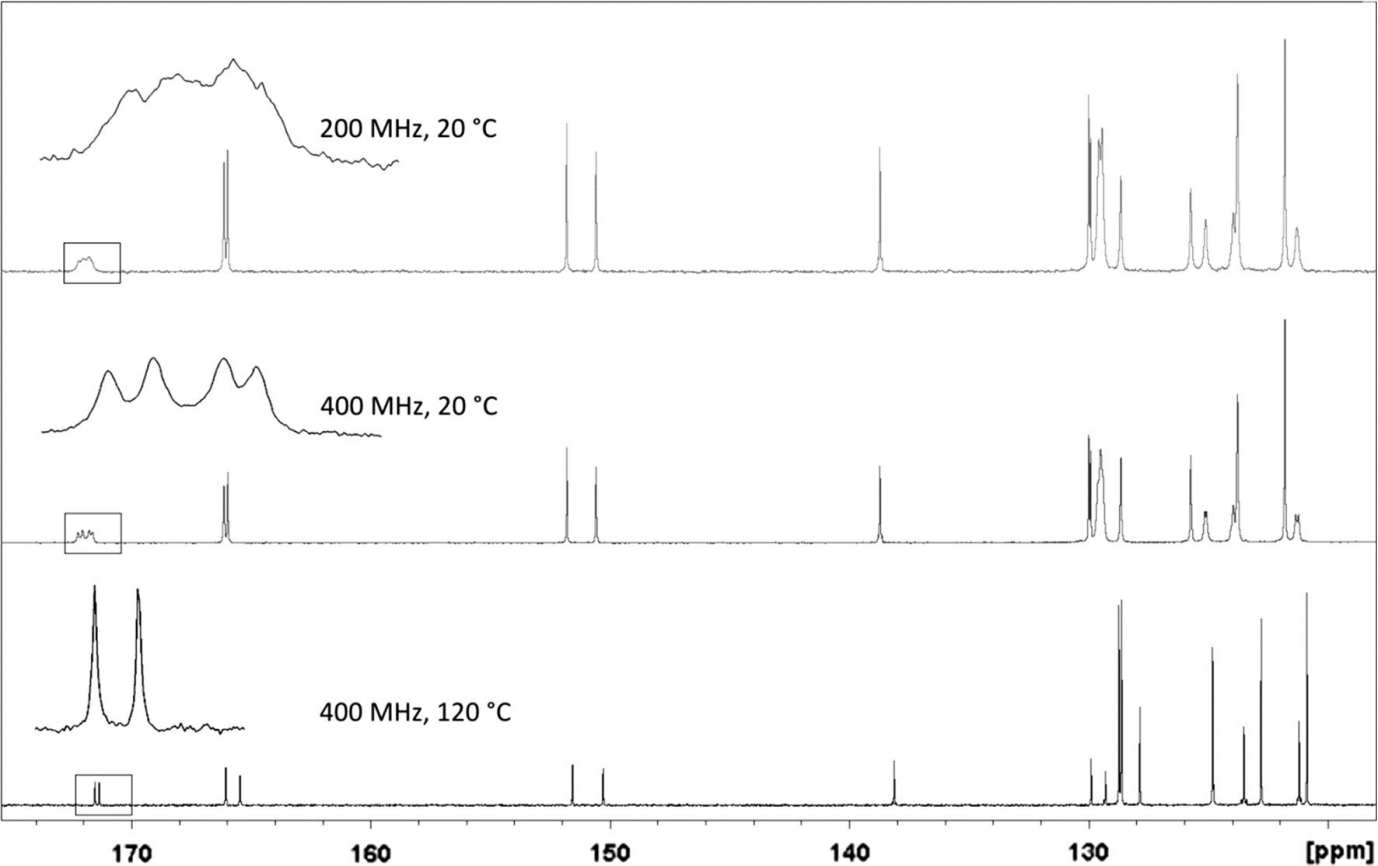
^13^C NMR of compound **5c**, recorded under different conditions

While 2,4,6-trisubstituted 1,3,5-triazines present rather ordinary proton NMR spectra, a noteworthy aspect became apparent in ^13^C spectra: carbons C4 and C6, such as in compound **5c**, (Fig. 5; i.e., those adjacent to the aryloxy groups) appeared at low field in the 170–173 ppm range relative to TMS. They gave rather broad and also low intensity signals (Fig. 6 top) or were not discernable as distinct peaks, in which case they emerged only as a small bulge in the spectrum. This is true for the magnetic field strength of the instrument that was used to measure most of the samples (4.7 T, corresponding to 200 MHz of proton resonance frequency).

In many cases, the total number of carbon signals of the triazine compounds bearing two aryloxy and one phenylamino moiety showed more carbon signals than was expected. What is more, additional peak splitting was observed when the same sample was measured using higher resolution and field strength (400 MHz, compound **5c**, Fig. 6 middle vs. top).

**Fig. 7:**
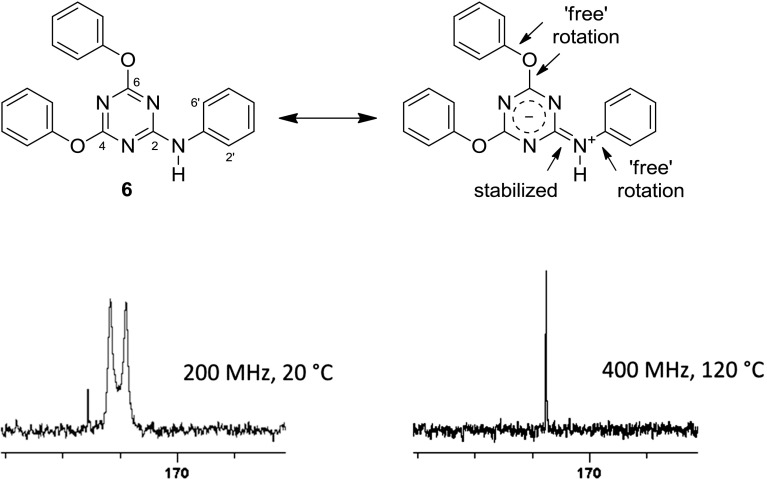
Low- versus high-temperature NMR of compound **6**

Thus, it seemed that in solution different long-lived (on the NMR time scale) conformers were present to give rise to more signals than expected. To test this hypothesis, high-temperature ^13^C spectra (at 120 °C) were recorded. Indeed, under such conditions, fewer carbon signals were observed, confirming that the compound must experience hindered internal rotation at room temperature (Fig. 6, bottom). To elucidate the situation further, the simpler compound **6** was also subjected to ^13^C NMR-measurement at 120 °C. The effect was particularly prominent in the case of carbon positions C4 and C6; they showed up as two broad signals at 171.8 and 172.3 ppm at room temperature, but also coalesced at 171.5 ppm as a sharp peak, making it very plausible that rotation around the C2–N bond was slowed down at room temperature. This can be explained by considering the highly electron-deficient triazine and the appropriate resonance contributor as in Fig. 7. C4 and C6 then adopt different relative conformations, i.e., *cis* and *trans* to carbons C2’ and C6’, respectively, as the C2–N bond displays partial double bond character. These conformations are stable long enough that carbon nuclei C4 and C6 become magnetically different and are visible on the NMR time scale as separate signals.

**Fig. 8:**
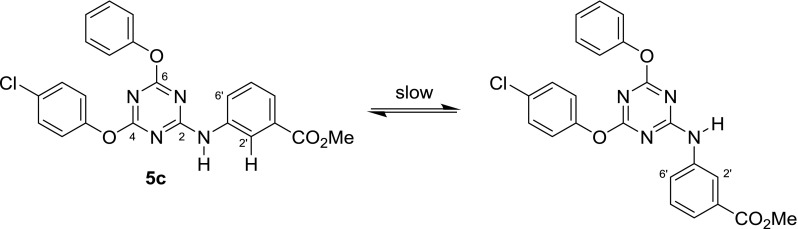
Rotamers of compound **5c**

In addition, if these two carbons are also chemically unequal, the two stable conformers become different rotamers, and the expected two signals split into four (as with **5c**, Figs. 6, 8). There is no *cis*/*trans* isomerism of this sort regarding carbon C2 itself, and this nucleus is, therefore, unaffected by peak splitting.

There are no additional peaks in the spectra of compounds where the NH is replaced by some other atom, such as sulfur in **7**. It, therefore, appears evident that the hindered rotation around this carbon–nitrogen bond is responsible for this isomerism as observed with compounds like **5c**.

#### 2,4-Dichloro-6-phenoxy-1,3,5-triazine (**2a**, C_9_H_5_Cl_2_N_3_O)

2,4,6-Trichloro-1,3,5-triazine (1.225 g, 6.64 mmol, 1.00 equiv.), dissolved in 14 cm^3^ THF, was cooled to − 15 °C (ice/salt bath), and then, 0.858 g DIPEA (6.64 mmol, 1.00 equiv.) and 0.625 g phenol (6.64 mmol, 1.00 equiv.) were added slowly while stirring. The reaction was kept at that temperature for 30 min, after that the temperature was raised to 0 °C and stirring was continued for another 3.75 h. Then, the mixture was warmed to r. t. within 30 min. The formed DIPEA-hydrochloride was removed by filtration and washed with additional THF (2 × 10 cm^3^). The solution was evaporated to give the solid crude product. It was again dissolved in 100 cm^3^ EtOAc and this organic solution was washed with saturated NaHCO_3_ solution (2 × 100 cm^3^), 100 cm^3^ 5% KHSO_4_, and 100 cm^3^ brine. After drying over Na_2_SO_4_, the solvent was removed and the product dried in vacuo without further purification. Yield: 1.521 g (95%); appearance: colorless solid; m.p.: 115–116 °C (lit.: 111.5–113.8 °C) [52]; *R*_*f*_ = 0.50 (hexane/EtOAc, 5:1); ^1^H NMR (CDCl_3_, 200 MHz): *δ* = 7.13–7.23 (m, 2H, H2’ & H6’), 7.28–7.39 (m, 1H, H4’), 7.41–7.53 (m, 2H, H3’ & H5’) ppm; ^13^C NMR (CDCl_3_, 50 MHz): *δ* = 121.1 (d, C2’ & C6’), 127.1 (d, C4’), 130.1 (d, C3’ & C5’), 151.2 (s, C1’), 171.3 (s, C6), 173.2 (s, C2 & C4) ppm; GC–MS (EI, 70 eV): *t*_*R*_ = 8.08 min; *m/z* (%) = 243 (M^+^, 10), 241 (M^+^, 15), 208 (32), 206 (100), 143 (11).

#### 2,4-Dichloro-6-(4-chlorophenoxy)-1,3,5-triazine (**2b**, C_9_H_4_Cl_3_N_3_O)

The reaction was performed in an 8 cm^3^ glass vial, using a cryo or thermo block. 2,4,6-Trichlorotriazine (369 mg, 2.00 mmol, 1.00 equiv.) was dissolved in 3.0 cm^3^ THF and cooled to − 35 °C. DIPEA (349 mg, 2.70 mmol, 1.35 equiv.) was added to 257 mg 4-chlorophenol (2.00 mmol, 1.00 equiv.), dissolved in 1.6 cm^3^ THF; the mixture was also cooled to − 35 °C, before this solution was added slowly to the trichlorotriazine solution while stirring. To ensure complete transfer, another 1.6 cm^3^ of THF, cooled to − 35 °C, was used to flush all phenol into the reaction mixture. Stirring was continued at this temperature for 3 h, then at r. t. for 27 h. H_2_O (10 cm^3^) was then added to the reaction mixture and the compound was extracted with EtOAc (2 × 10 cm^3^), followed by washing with 10 cm^3^ brine. The solution was concentrated in vacuo and the compound [53] was purified by column chromatography (MPLC, 90 g silica, 90 cm^3^ min^−1^ flow rate, LP with 2% EtOAc), and finally dried in vacuo. Yield: 243 mg (44%); appearance: colorless solid; m.p.: 105–108 °C; *R*_*f*_ = 0.51 (hexane/EtOAc, 5:1); ^1^H NMR (CDCl_3_, 200 MHz): *δ* = 7.03–7.17 (m, 2H, H2’ & H6’), 7.32–7.46 (m, 2H, H3’ & H5’) ppm; ^13^C NMR (CDCl_3_, 50 MHz): *δ* = 122.5 (d, C2’ & C6’), 130.1 (d, C3’ & C5’), 132.6 (s, C4’), 149.5 (s, C1’), 171.0 (s, C6), 173.2 (s, C2 & C4) ppm; GC–MS (EI, 70 eV): *t*_*R*_ = 9.16 min; *m/z* (%) = 277 (M^+^, 12), 275 (M^+^, 15), 244 (10), 242 (62), 241 (12), 240 (100), 177 (15), 111 (24).

#### 2-Chloro-4,6-diphenoxy-1,3,5-triazine (**3a**, C_15_H_10_ClN_3_O_2_)

2,4,6-Trichloro-1,3,5-triazine (1.248 g, 6.77 mmol, 1.00 equiv.), dissolved in 7 cm^3^ THF, was cooled to − 30 °C; then, a solution of 0.637 g phenol (6.77 mmol, 1.00 equiv.) and 0.874 g DIPEA (6.77 mmol, 1.00 equiv.) in 7 cm^3^ THF was added dropwise over 10 min while stirring. The reaction was kept at − 35 to − 30 °C for another 30 min and subsequently at 0 °C for 5 h. Then, another solution of 0.637 g phenol (6.77 mmol, 1.00 equiv.) and 0.874 g DIPEA (6.77 mmol, 1.00 equiv.) in 7 cm^3^ THF was added dropwise over 15 min and the mixture was warmed slowly to r. t. After 48 h, the reaction temperature was raised to 50 °C and stirring was continued for 4.5 h. The formed DIPEA-hydrochloride was removed by filtration and washed with additional THF (30 cm^3^). The solution was evaporated to give the solid crude product. It was again dissolved in 100 cm^3^ EtOAc and this organic solution was washed with Na_2_CO_3_ solution, 0.5 M HCl, as well as brine (2 × 100 cm^3^ for each) and subsequently dried over Na_2_SO_4_. The solvent was removed and the crude material was re-crystallized from *n*-hexane/toluene (35 cm^3^/7 cm^3^), collected by filtration, washed with 20 cm^3^ *n*-hexane, and dried in vacuo. Yield: 1.584 g (78%); appearance: colorless crystals; m.p.: 120.5–121 °C (lit.: 119–121 °C) [54]; *R*_*f*_ = 0.39 (hexane/EtOAc, 5:1); ^1^H NMR (CDCl_3_, 200 MHz): *δ* = 7.10–7.19 (m, 4H, H2’ & H6’ & H2” & H6”), 7.22–7.32 (m, 2H, H4’ & H4”), 7.34–7.46 (m, 4H, H3’ & H5’ & H3” & H5”) ppm; ^13^C NMR (CDCl_3_, 50 MHz): *δ* = 121.2 (d, C2’ & C6’), 126.5 (d, C4’), 129.7 (d, C3’ & C5’), 151.3 (s, C1’), 172.4 (s, C4 & C6), 173.8 (s, C2) ppm; GC–MS (EI, 70 eV): *t*_*R*_ = 11.45 min; *m/z* (%) = 301 (M^+^, 11), 300 (M^+^, 10), 299 (M^+^, 33), 298 (16), 264 (57), 208 (32), 207 (11), 206 (100), 180 (12), 145 (15), 140 (10), 138 (31), 121 (20).

### Synthesis of (4,6-disubstituted-1,3,5-triazin-2-yl)aminobenzoic acids **4a**–**4k**—general procedure A

The reaction was performed in an 8 cm^3^ glass vial, using a thermo block. Formic acid (4.0 cm^3^) was added to 1,1-dimethylethyl aminobenzoate (1.00 equiv.). This mixture was briefly sonicated until the starting material was dissolved and stirred at the specified temperature until TLC showed complete conversion. The content of the reaction vial was transferred to a round bottom flask and the vial was flushed with a few cm^3^ of CH_2_Cl_2_. Then, the solvents were removed under reduced pressure, 10 cm^3^ Et_2_O was added to facilitate the removal of residual formic acid, the solution evaporated again and finally dried in vacuo.

#### 3-[(4,6-Diphenoxy-1,3,5-triazin-2-yl)amino]benzoic acid (**4a**, C_22_H_16_N_4_O_4_)

Prepared according to general procedure A using 46 mg 1,1-dimethylethyl aminobenzoate **8a** (0.10 mmol, 1.00 equiv.); 50 °C for 1 h; compound **4a** is literature-known [31, 55]. Yield: 40 mg (quant.); appearance: off-white solid; m.p.: 267.5–270 °C; *R*_*f*_ = 0.57 (hexane/EtOH, 1:1); ^1^H NMR (DMSO-*d*_*6*_, 200 MHz): *δ* = 7.14–7.35 (m, 7H), 7.38–7.52 (m, 4H), 7.56 (d, *J*_3_ = 7.7 Hz, 1H), 7.76 (d, *J*_3_ = 8.3 Hz, 1H), 8.03 (s, 1H, H2’), 10.40 (s, 1H, NH), 12.98 (bs, 1H, CO_2_H) ppm; ^13^C NMR (DMSO-*d*_*6*_, 50 MHz): *δ* = 121.5 (d, C2’), 121.8 (d, C2” & C2’” & C6” & C6’”), 124.1 (d, C4’), 124.8 (d, C6’), 125.7 (d, C4” & C4’”), 128.5 (d, C5’), 129.6 (d, C3” & C3’” & C5” & C5’”), 131.2 (s, C3’), 138.6 (s, C1’), 151.8 (s, C1”, C1’”), 166.2 (s, C2), 167.1 (s, CO_2_), 171.8 & 172.2 (bs, C4 & C6, rotamers) ppm.

#### 3-[[4-(4-Chlorophenoxy)-6-phenoxy-1,3,5-triazin-2-yl]amino]benzoic acid (**4b**, C_22_H_15_ClN_4_O_4_)

Prepared according to general procedure A using 49 mg 1,1-dimethylethyl aminobenzoate **8b** (0.10 mmol, 1.00 equiv.); 50 °C for 1 h. Yield: 44 mg (quant.); appearance: beige solid; m.p.: 244–247 °C; *R*_*f*_ = 0.63 (hexane/EtOH, 1:1); ^1^H NMR (DMSO-*d*_*6*_, 200 MHz): *δ* = 7.14–7.38 (m, 6H), 7.38–7.63 (m, 5H), 7.72 (d, *J*_3_ = 8.4 Hz, 1H), 8.04 (s, 1H, H2’), 10.42 (s, 1H, NH), 12.91 (bs, 1H, CO_2_H) ppm; ^13^C NMR (DMSO-*d*_*6*_, 50 MHz): *δ* = 121.5 (d, C2’), 121.8 (d, C2’” & C6’”), 123.8 (d, C2” & C6”), 124.2 (d, C4’), 124.8 (d, C6’), 125.8 (d, C4’”), 128.5 (d, C5’), 129.5 (d, C3”* & C5”*), 129.6 (d, C3’”* & C5’”*), 129.9 (s, C4”), 131.2 (s, C3’), 138.5 (s, C1’), 150.6 (s, C1”), 151.8 (s, C1’”), 166.1 (s, C2), 167.0 (s, CO_2_), 171.3–172.4 (C4 & C6) ppm.

#### 3-[[4-(3-Chlorophenoxy)-6-phenoxy-1,3,5-triazin-2-yl]amino]benzoic acid (**4c**, C_22_H_15_ClN_4_O_4_)

Prepared according to general procedure A using 49 mg 1,1-dimethylethyl aminobenzoate **8c** (0.10 mmol, 1.00 equiv.); 50 °C for 1 h. Yield: 44 mg (quant.); appearance: off-white solid; m.p.: 227–230 °C; *R*_*f*_ = 0.59 (hexane/EtOH, 1:1); ^1^H NMR (DMSO-*d*_*6*_, 200 MHz): *δ* = 7.16–7.53 (m, 10H), 7.58 (d, *J*_3_ = 7.7 Hz, 1H), 7.75 (d, *J*_3_ = 7.9 Hz, 1H), 8.04 (s, 1H, H2’), 10.45 (s, 1H, NH), 12.97 (bs, 1H, CO_2_H) ppm; ^13^C NMR (DMSO-*d*_*6*_, 50 MHz): *δ* = 120.8 (d, C6”), 121.5 (d, C2’), 121.8 (d, C2’” & C6’”), 122.4 (d, C2”), 124.3 (d, C4’), 124.8 (d, C6’), 125.8 (d, C4’”), 125.9 (d, C4”), 128.5 (d, C5’), 129.6 (d, C3’” & C5’”), 131.0 (d, C5”), 131.4 (s, C3’), 133.4 (s, C3”), 138.5 (s, C1’), 151.8 (s, C1’”), 152.4 (s, C1”), 166.2 (s, C2), 167.1 (s, CO_2_*), 171.3–172.3 (C4 & C6, not resolved) ppm.

#### 3-[[4-(2-Chlorophenoxy)-6-phenoxy-1,3,5-triazin-2-yl]amino]benzoic acid (**4d**, C_22_H_15_ClN_4_O_4_)

Prepared according to general procedure A using 49 mg 1,1-dimethylethyl aminobenzoate **8d** (0.10 mmol, 1.00 equiv.); 50 °C for 1 h. Yield: 44 mg (quant.); appearance: colorless crystals; m.p.: 249.5–251.5 °C; *R*_*f*_ = 0.60 (hexane/EtOH, 1:1); ^1^H NMR (DMSO-*d*_*6*_, 200 MHz): *δ* = 7.13–7.51 (m, 9H), 7.52–7.75 (m, 3H), 7.99 (s, 1H, H2’), 10.49 (s, 1H, NH), 12.93 (bs, 1H, CO_2_H) ppm; ^13^C NMR (DMSO-*d*_*6*_, 50 MHz): *δ* = 121.6 (d, C2’), 121.8 (d, C2’” & C6’”), 124.3 (d, C6”*), 124.8 (d, C6’), 125.8 (d, C4’”), 126.0 (s, C2”), 127.5 (d, C4”*), 128.5 (d, C5”*), 128.6 (d, C5’*), 129.7 (d, C3’” & C5’”), 130.3 (d, C3”), 131.3 (s, C3’), 138.5 (s, C1’), 147.7 (s, C1”), 151.8 (s, C1’”), 166.2 (s, C2), 167.1 (s, CO_2_), 171.2–172.5 (C4 & C6, not resolved) ppm, one d signal missing possibly due to overlap.

#### 3-[[4-[4-(1,1-Dimethylethyl)phenoxy]-6-phenoxy-1,3,5-triazin-2-yl]amino]benzoic acid (**4e**, C_26_H_24_N_4_O_4_)

Prepared according to general procedure A using 51 mg 1,1-dimethylethyl aminobenzoate **8e** (0.10 mmol, 1.00 equiv.); 45 °C for 1 h. Yield: 46 mg (quant.); appearance: off-white solid; m.p.: 245–247 °C; *R*_*f*_ = 0.67 (hexane/EtOH, 1:1); ^1^H NMR (DMSO-*d*_*6*_, 200 MHz): *δ* = 1.30 (s, 9H, (CH_3_)_3_), 7.10–7.33 (m, 6H), 7.37–7.51 (m, 4H), 7.57 (d, *J*_3_ = 7.7 Hz, 1H), 7.75 (d, *J*_3_ = 8.7 Hz, 1H), 8.05 (s, 1H, H2’), 10.40 (s, 1H, NH), 12.92 (bs, 1H, CO_2_H) ppm; ^13^C NMR (DMSO-*d*_*6*_, 50 MHz): *δ* = 31.3 (q, C(CH_3_)_3_), 34.3 (s, C(CH_3_)_3_), 121.2 (d, C2” & C6”), 121.6 (d, C2’), 121.8 (d, C2’” & C6’”), 124.1 (d, C4’), 124.8 (d, C6’), 125.7 (d, C4’”), 126.3 (d, C3” & C5”), 128.4 (d, C5’), 129.6 (d, C3’” & C5’”), 131.2 (s, C3’), 138.7 (s, C1’), 148.0 (s, C4”*), 149.5 (s, C1”*), 151.8 (s, C1’”), 166.2 (s, C2), 167.1 (s, CO_2_), 171.6–172.7 (C4 & C6, not resolved) ppm.

#### 3-[[4-(4-Methoxyphenoxy)-6-phenoxy-1,3,5-triazin-2-yl]amino]benzoic acid (**4f**, C_23_H_18_N_4_O_5_)

Prepared according to general procedure A using 49 mg 1,1-dimethylethyl aminobenzoate **8f** (0.10 mmol, 1.00 equiv.); 50 °C for 1 h. Yield: 43 mg (quant.); appearance: off-white solid; m.p.: 233–236.5 °C; *R*_*f*_ = 0.65 (hexane/EtOH, 1:1); ^1^H NMR (DMSO-*d*_*6*_, 200 MHz): *δ* = 3.77 (s, 3H, OCH_3_), 6.97 (d, *J*_3_ = 9.0 Hz, 2H, H3” & H5”), 7.13–7.34 (m, 6H), 7.39–7.51 (m, 2H), 7.56 (d, *J*_3_ = 7.6 Hz, 1H), 7.74 (d, *J*_3_ = 8.2 Hz, 1H), 8.04 (s, 1H, H2’), 10.37 (s, 1H, NH), 12.93 (bs, 1H, CO_2_H) ppm; ^13^C NMR (DMSO-*d*_*6*_, 50 MHz): *δ* = 55.5 (q, OCH_3_), 114.5 (d, C3” & C5”), 121.5 (d, C2’), 121.8 (d, C2’” & C6’”), 122.7 (d, C2” & C6”), 124.1 (d, C4’), 124.8 (d, C6’), 125.7 (d, C4’”), 128.5 (d, C5’), 129.6 (d, C3’” & C5’”), 131.3 (s, C3’), 138.6 (s, C1’), 145.2 (s, C1”), 151.8 (s, C1’”), 156.9 (s, C4”), 166.2 (s, C2), 167.1 (s, CO_2_), 171.3–172.7 (C4 & C6, not resolved) ppm.

#### 3-[[4-(4-Cyanophenoxy)-6-phenoxy-1,3,5-triazin-2-yl]amino]benzoic acid (**4g**, C_23_H_15_N_5_O_4_)

Prepared according to general procedure A using 48 mg 1,1-dimethylethyl aminobenzoate **8g** (0.10 mmol, 1.00 equiv.); 45 °C for 1 h. Yield: 43 mg (quant.); appearance: colorless solid; m.p.: 150 °C (decomp.); *R*_*f*_ = 0.65 (hexane/EtOH, 1:1); ^1^H NMR (DMSO-*d*_*6*_, 200 MHz): *δ* = 7.16–7.35 (m, 4H), 7.37–7.77 (m, 6H), 7.94 (d, *J*_3_ = 8.1 Hz, 2H, H3” & H5”), 8.06 (s, 1H, H2’), 10.48 (s, 1H, NH) ppm, CO_2_H not visible; ^13^C NMR (DMSO-*d*_*6*_, 50 MHz): *δ* = 108.6 (s, C4”), 118.5 (s, CN), 121.66 (d, C2’), 121.76 (d, C2’” & C6’”), 123.3 (d, C2” & C6”), 124.4 (d, C4’), 124.8 (d, C6’), 125.8 (d, C4’”), 128.5 (d, C5’), 129.6 (d, C3’” & C5’”), 131.6 (s, C3’), 134.0 (d, C3” & C5”), 138.4 (s, C1’), 151.6 (s, C1’”), 155.2 (s, C1”), 166.2 (s, C2), 167.1 (s, CO_2_), 170.9–172.2 (C4 & C6, not resolved) ppm.

#### 3-[[4-(3-Nitrophenoxy)-6-phenoxy-1,3,5-triazin-2-yl]amino]benzoic acid (**4h**, C_22_H_15_N_5_O_6_)

Prepared according to general procedure A using 40 mg 1,1-dimethylethyl aminobenzoate **8h** (0.08 mmol, 1.00 equiv.); 45 °C for 1 h. Yield: 36 mg (quant.); appearance: beige solid; m.p.: 225–228 °C; *R*_*f*_ = 0.51 (hexane/EtOH, 1:1); ^1^H NMR (DMSO-*d*_*6*_, 200 MHz): *δ* = 7.16–7.33 (m, 4H), 7.34–7.50 (m, 2H), 7.57 (d, *J*_3_ = 7.7 Hz, 1H), 7.63–7.84 (m, 3H), 8.06 (s, 1H, H2’), 8.15 (d, *J*_3_ = 7.5 Hz, 1H), 8.22 (m, 1H, H2”), 10.49 (s, 1H, NH), 12.83 (bs, 1H, CO_2_H) ppm; ^13^C NMR (DMSO-*d*_*6*_, 50 MHz): *δ* = 117.5 (d, C2”), 120.7 (d, C4”), 121.5 (d, C2’), 121.8 (d, C2’” & C6’”), 124.4 (d, C4’), 124.8 ((d, C6’), 125.8 (d, C4’”), 128.5 (d, C6”*), 128.9 (d, C5’*), 129.6 (d, C3’” & C5’”), 130.8 (d, C5”), 131.3 (s, C3’), 138.5 (s, C1’), 148.4 (s, C3”), 151.8 (s, C1’”*), 152.0 (s, C1”*), 166.2 (s, C2), 167.1 (s, CO_2_), 171.2–172.5 (C4 & C6, not resolved) ppm.

#### 3-[[4-(4-Formyl-2-methoxyphenoxy)-6-phenoxy-1,3,5-triazin-2-yl]amino]benzoic acid (**4i**, C_24_H_18_N_4_O_6_)

Prepared according to general procedure A using 39 mg 1,1-dimethylethyl aminobenzoate **8i** (0.075 mmol, 1.00 equiv.); 45 °C for 1 h. Yield: 34 mg (quant.); appearance: pale yellow solid; m.p.: 217.5–220 °C; *R*_*f*_ = 0.62 (hexane/EtOH, 1:1); ^1^H NMR (DMSO-*d*_*6*_, 200 MHz): *δ* = 3.86 (s, 3H, OCH_3_), 7.12–7.33 (m, 4H), 7.35–7.75 (m, 7H), 8.00 (s, 1H, H2’), 10.00 (s, 1H, CHO), 10.44 (s, 1H, NH) ppm, CO_2_H not visible; ^13^C NMR (DMSO-*d*_*6*_, 50 MHz): *δ* = 56.1 (q, OCH_3_), 112.3 (d, C3”), 121.5 (d, C2’), 121.8 (d, C2’” & C6’”), 123.7 (d, C5”*), 123.8 (d, C4’*), 124.3 (d, C6”*), 124.7 (d, C6’), 125.8 (d, C4’”), 128.5 (d, C5’), 129.7 (d, C3’” & C5’”), 131.4 (s, C3’), 135.1 (s, C4”), 138.5 (s, C1’), 145.3 (s, C1”*), 151.70 (s, C2”*), 151.75 (s, C1’”*), 166.1 (s, C2), 167.1 (s, CO_2_*), 171.2–172.7 (C4 & C6, not resolved), 192.1 (d, CHO) ppm.

#### 3-[[4-(4-Formyl-2-methoxyphenoxy)-6-(4-methoxyphenoxy)-1,3,5-triazin-2-yl]amino]benzoic acid (**4j**, C_25_H_20_N_4_O_7_)

Prepared according to general procedure A using 37 mg 1,1-dimethylethyl aminobenzoate **8j** (0.068 mmol, 1.00 equiv.); 45 °C for 1.25 h. Yield: 33 mg (quant.); appearance: colorless solid; m.p.: 180–182 °C; *R*_*f*_ = 0.59 (hexane/EtOH, 1:1); ^1^H NMR (DMSO-*d*_*6*_, 200 MHz): *δ* = 3.76 (s, 3H, C4’”OCH_3_), 3.86 (s, 3H, C2”OCH_3_), 6.96 (d, *J*_3_ = 9.1 Hz, 2H, H3’” & H5’”), 7.11–7.29 (m, 3H), 7.42–7.83 (m, 5H), 8.02 (s, 1H, H2’), 10.00 (s, 1H, CHO), 10.42 (s, 1H, NH), 12.92 (bs, 1H, CO_2_H) ppm; ^13^C NMR (DMSO-*d*_*6*_, 100 MHz): *δ* = 55.9 (q, C4’”OCH_3_), 56.5 (q, C2”OCH_3_), 112.8 (d, C3”), 114.9 (d, C3’” & C5’”), 122.4 (d), 123.1 (d, C2’” & C6’”), 124.1 (d), 125.0 (d), 127.6 (d), 135.4 (s, C4”), 137.9 (s, C1’), 139.7 (s), 145.7 (s, C1”*), 145.8 (s, C1’”*), 152.2 (s, C2”), 157.3 (s, C4’”), 166.6 (s), 171.4–173.0 (C4 & C6, not resolved), 192.6 (d, CHO) ppm, one s and two d signals missing possibly due to overlap.

#### 3-[[4-(4-Cyanophenoxy)-6-(4-methoxyphenoxy)-1,3,5-triazin-2-yl]amino]benzoic acid (**4k**, C_24_H_17_N_5_O_5_)

Prepared according to general procedure A using 31 mg 1,1-dimethylethyl aminobenzoate **8k** (0.06 mmol, 1.00 equiv.); 45 °C for 1 h. Yield: 27 mg (quant.); appearance: colorless solid; m.p.: 240.5–242.5 °C; *R*_*f*_ = 0.48 (hexane/EtOH, 1:1); ^1^H NMR (DMSO-*d*_*6*_, 200 MHz): *δ* = 3.77 (s, 3H, OCH_3_), 6.97 (d, *J*_3_ = 9.0 Hz, 2H, H3’” & H5’”), 7.11–7.33 (m, 3H), 7.46–7.80 (m, 4H), 7.94 (d, *J*_3_ = 8.3 Hz, 2H, H3” & H5”), 8.06 (s, 1H, H2’), 10.44 (s, 1H, NH) ppm, CO_2_H not visible; ^13^C NMR (DMSO-*d*_*6*_, 50 MHz): *δ* = 55.5 (q, OCH_3_), 108.6 (s, C4”), 114.5 (d, C3’” & C5’”), 118.5 (s, CN), 121.7 (d, C2’), 122.6 (d, C2’” & C6’”), 123.3 (d, C2” & C6”), 124.4 (d, C4’), 124.9 (d, C6’), 128.6 (d, C5’), 131.4 (s, C3’), 134.2 (d, C3” & C5”), 138.5 (s, C1’), 145.2 (s, C1’”), 155.2 (s, C1”), 156.9 (s, C4’”), 166.2 (s, C2), 167.1 (s, CO_2_) ppm, C4 & C6 not visible.

### Synthesis of methyl and ethyl (4,6-disubstituted-1,3,5-triazin-2-yl)aminobenzoates—general procedure B

The reaction was performed in an 8 cm^3^ glass vial, using a cryo or thermoblock. DIPEA (1.10 equiv.) was added to the phenolic compound R^1^PhOH (1.00 equiv.) dissolved in 0.5 cm^3^ THF. This solution was cooled to − 35 °C and then added dropwise to a solution of 2,4,6-trichlorotriazine (1.00 equiv.) in 2.0 cm^3^ THF at − 35 °C while stirring. To ensure complete transfer, another 0.5 cm^3^ of THF was used to flush all phenolic compound into the reaction mixture. Stirring was continued (step 1) at this temperature until TLC indicated complete conversion or no significant change in reaction composition. Then, DIPEA (1.60 equiv.) was added to a solution of phenol R^2^PhOH (1.00 equiv.) in 0.5 cm^3^ THF, and this mixture was added to the reaction. To ensure complete transfer, another 0.5 cm^3^ of THF was used to flush all phenolic compounds into the reaction mixture. Stirring was continued (step 2) and checked by TLC. Then, DIPEA (1.50 equiv.), followed by alkyl aminobenzoate (1.15 equiv.), was added directly to the reaction mixture. The reaction was then continued (step 3) and checked by TLC. For work-up, 15 cm^3^ CH_2_Cl_2_ was added to the reaction mixture followed by 15 cm^3^ 1 N HCl_aq_, and the compound was extracted. The layers were separated; the organic phase was washed with water (2 × 15 cm^3^) and then concentrated in vacuo. Further work-up and purification procedures are given at the respective examples.

#### Ethyl 4-[(4,6-diphenoxy-1,3,5-triazin-2-yl)amino]benzoate (**5a**, C_24_H_20_N_4_O_4_)

Prepared according to general procedure B using for step 1: 277 mg 2,4,6-trichlorotriazine (1.50 mmol, 1.00 equiv.), 141 mg phenol (1.50 mmol, 1.00 equiv.), 213 mg DIPEA (1.65 mmol, 1.10 equiv.); − 35 °C for 6 h; step 2: 141 mg phenol (1.50 mmol, 1.00 equiv.), 310 mg DIPEA (2.40 mmol, 1.60 equiv.); 35 °C for 46 h; step 3: 285 mg ethyl 4-aminobenzoate (1.73 mmol, 1.15 equiv.), 291 mg DIPEA (2.25 mmol, 1.50 equiv.); 40 °C for 42 h. After the work-up procedure described in general procedure B, 1 cm^3^ diethyl ether and 2 cm^3^ LP were added to induce precipitation and facilitate evaporation to dryness. The crude material was dissolved in a mixture of refluxing CHCl_3_/LP (1: 2, 12 cm^3^) and 8 cm^3^ *n*-hexane was added. After crystallization overnight, the compound was isolated by filtration, washed with 40 cm^3^ LP, and dried in vacuo. Yield: 611 mg (95%); appearance: colorless solid; m.p.: 159–161 °C; *R*_*f*_ = 0.54 (hexane/EtOAc, 2:1); ^1^H NMR (DMSO-*d*_*6*_, 200 MHz): *δ* = 1.28 (t, *J*_3_ = 7.1 Hz, 3H, CH_3_), 4.25 (q, *J*_3_ = 7.1 Hz, 2H, CH_2_), 7.26–7.35 (m, 6H), 7.45–7.57 (m, 6H), 7.63–7.74 (m, 2H), 10.57 (s, 1H, NH) ppm; ^13^C NMR (DMSO-*d*_*6*_, 50 MHz): *δ* = 14.1 (q, CH_3_), 60.4 (t, CH_2_), 119.4 (d, C2’ & C6’), 121.9 (d, C2” & C2’” & C6” & C6’”), 123.9 (s, C4’), 125.8 (d, C4” & C4’”), 129.60 (C3’ & C5’), 129.63 (d, C3” & C3’” & C5” & C5’”), 143.0 (s, C1’), 151.9 (s, C1” & C1’”), 165.3 (s, CO_2_), 166.0 (s, C2), 172.1 (s, C4 & C6, no rotamers visible individually) ppm.

#### Methyl 3-[(4,6-diphenoxy-1,3,5-triazin-2-yl)amino]benzoate (**5b**, C_23_H_18_N_4_O_4_)

Prepared according to general procedure B using for step 1: 277 mg 2,4,6-trichlorotriazine (1.50 mmol, 1.00 equiv.), 141 mg phenol (1.50 mmol, 1.00 equiv.), 213 mg DIPEA (1.65 mmol, 1.10 equiv.); − 35 °C for 6 h; step 2: 141 mg phenol (1.50 mmol, 1.00 equiv.), 310 mg DIPEA (2.40 mmol, 1.60 equiv.); 35 °C for 46 h; step 3: 261 mg methyl 3-aminobenzoate (1.73 mmol, 1.15 equiv.), 291 mg DIPEA (2.25 mmol, 1.50 equiv.); 35 °C for 26 h. After the work-up procedure described in general procedure B, the compound crystallized; the material was then dissolved in 10 cm^3^ refluxing chloroform and 10 cm^3^ *n*-hexane was added to the boiling mixture, which caused the compound to precipitate. After crystallization overnight, the compound was isolated by filtration, washed with 30 cm^3^ LP and dried in vacuo. Yield: 523 mg (84%); appearance: off-white powder; m.p.: 198–199.5 °C; *R*_*f*_ = 0.50 (hexane/EtOAc, 2:1); ^1^H NMR (DMSO-*d*_*6*_, 200 MHz): *δ* = 3.81 (s, 3H, CH_3_), 7.18–7.31 (m, 7H), 7.41–7.48 (m, 4H), 7.56 (d, *J*_3_ = 7.8 Hz, 1H), 7.75 (d, *J*_3_ = 7.9 Hz, 1H), 8.03 (s, 1H, H2’), 10.42 (s, 1H, NH) ppm; ^13^C NMR (DMSO-*d*_*6*_, 50 MHz): *δ* = 52.2 (q, CH_3_), 121.2 (d, C2’), 121.8 (d, C2” & C2’” & C6” & C6’”), 123.9 (d, C4’), 125.1 (d, C6’), 125.7 (d, C4” & C4’”), 128.7 (d, C5’), 129.6 (d, C3” & C3’” & C5” & C5’”), 130.0 (s, C3’), 138.9 (s, C1’), 151.8 (s, C1”, C1’”), 166.0 (s, C2*), 166.2 (s, CO_2_*), 171.8 & 172.2 (bs, C4 & C6, rotamers) ppm.

#### Methyl 3-[[4-(4-chlorophenoxy)-6-phenoxy-1,3,5-triazin-2-yl]amino]benzoate (**5c**, C_23_H_17_ClN_4_O_4_)

Prepared according to general procedure B using for step 1: 277 mg 2,4,6-trichlorotriazine (1.50 mmol, 1.00 equiv.), 141 mg phenol (1.50 mmol, 1.00 equiv.), 213 mg DIPEA (1.65 mmol, 1.10 equiv.); − 35 °C for 6 h; step 2: 193 mg 4-chlorophenol (1.50 mmol, 1.00 equiv.), 310 mg DIPEA (2.40 mmol, 1.60 equiv.); 35 °C for 46 h; step 3: 261 mg methyl 3-aminobenzoate (1.73 mmol, 1.15 equiv.), 291 mg DIPEA (2.25 mmol, 1.50 equiv.); 35 °C for 26 h. After the work-up procedure described in general procedure B, approx. 5 cm^3^ LP was added to induce precipitation and facilitate evaporation to dryness. The crude material was dissolved in 5.5 cm^3^ refluxing CHCl_3_ and 5.5 cm^3^ *n*-hexane was added. The solution was briefly sonicated to facilitate crystallization and the compound was isolated by filtration, washed with 15 cm^3^ LP and dried in vacuo. Yield: 546 mg (81%); appearance: colorless powder; m.p.: 186.5–188 °C; *R*_*f*_ = 0.48 (hexane/EtOAc, 2:1); ^1^H NMR (DMSO-*d*_*6*_, 400 MHz): *δ* = 3.82 (s, 3H, CH_3_), 7.19–7.36 (m, 6H), 7.41–7.53 (m, 4H), 7.58 (d, *J*_3_ = 6 Hz, 1H), 7.76 (bs, 1H), 8.08 (s, 1H, H2’), 10.47 (s, 1H, NH) ppm; ^13^C NMR (DMSO-*d*_*6*_, 100 MHz, 120 °C): *δ* = 51.2 (q, CH_3_), 120.9 (d), 121.2 (d), 123.5 (d), 124.8 (d), 127.6 (d), 128.6 (d), 128.7 (d), 129.3 (s), 129.9 (s), 138.1 (s, C1’), 150.3 (s, C1”), 151.6 (s, C1’”), 164.5 (s, C2*), 166.1 (s, CO_2_*), 171.3 & 171.5 (s, C4 & C6) ppm; one d signal missing possibly due to overlap.

#### Methyl 3-[[4-(3-chlorophenoxy)-6-phenoxy-1,3,5-triazin-2-yl]amino]benzoate (**5d**, C_23_H_17_ClN_4_O_4_)

Prepared according to general procedure B using for step 1: 277 mg 2,4,6-trichlorotriazine (1.50 mmol, 1.00 equiv.), 141 mg phenol (1.50 mmol, 1.00 equiv.), 213 mg DIPEA (1.65 mmol, 1.10 equiv.); − 35 °C for 6 h; step 2: 193 mg 3-chlorophenol (1.50 mmol, 1.00 equiv.), 310 mg DIPEA (2.40 mmol, 1.60 equiv.); 35 °C for 46 h; step 3: 261 mg methyl 3-aminobenzoate (1.73 mmol, 1.15 equiv.), 291 mg DIPEA (2.25 mmol, 1.50 equiv.); 35 °C for 26 h. After the work-up procedure described in general procedure B, approx. 5 cm^3^ LP was added to induce precipitation and facilitate evaporation to dryness. The crude material was dissolved in 4 cm^3^ refluxing CHCl_3_ and 4 cm^3^ *n*-hexane was added. After cooling and crystallization were complete, the compound was isolated by filtration, washed with 20 cm^3^ LP, and dried in vacuo. Yield: 600 mg (89%); appearance: colorless powder; m.p.: 151.5–156 °C; *R*_*f*_ = 0.55 (hexane/EtOAc, 2:1); ^1^H NMR (DMSO-*d*_*6*_, 200 MHz): *δ* = 3.81 (s, 3H, CH_3_), 7.24–7.51 (m, 10H), 7.58 (d, *J*_3_ = 7.8 Hz, 1H), 7.77 (d, *J*_3_ = 8.3 Hz, 1H), 8.05 (t, *J* = 1.6 Hz, 1H, H2’), 10.48 (s, 1H, NH) ppm; ^13^C NMR (DMSO-*d*_*6*_, 50 MHz): *δ* = 52.2 (q, CH_3_), 120.7 (d, C6”), 121.3 (d, C2’), 121.8 (d, C2’” & C6’”), 122.4 (d, C2”), 124.0 (d, C4’), 125.1 (d, C6’), 125.7 (d, C4’”), 125.9 (d, C4”), 128.7 (d, C5’), 129.6 (d, C3’” & C5’”), 130.0 (s, C3’), 130.9 (d, C5”), 133.4 (s, C3”), 138.7 (s, C1’), 151.8 (s, C1’”), 152.4 (s, C1”), 166.0 (s, C2*), 166.2 (s, CO_2_*), 171.3–172.4 (C4 & C6, not resolved) ppm.

#### Methyl 3-[[4-(2-chlorophenoxy)-6-phenoxy-1,3,5-triazin-2-yl]amino]benzoate (**5e**, C_23_H_17_ClN_4_O_4_)

Prepared according to general procedure B using for step 1: 277 mg 2,4,6-trichlorotriazine (1.50 mmol, 1.00 equiv.), 141 mg phenol (1.50 mmol, 1.00 equiv.), 213 mg DIPEA (1.65 mmol, 1.10 equiv.); 35 °C for 6 h; step 2: 193 mg 2-chlorophenol (1.50 mmol, 1.00 equiv.), 310 mg DIPEA (2.40 mmol, 1.60 equiv.); 35 °C for 46 h; step 3: 261 mg methyl 3-aminobenzoate (1.73 mmol, 1.15 equiv.), 291 mg DIPEA (2.25 mmol, 1.50 equiv.); 35 °C for 26 h. After the work-up procedure described in general procedure B, approx. 5 cm^3^ LP was added to induce precipitation and facilitate evaporation to dryness. The crude material was dissolved in a refluxing mixture of CHCl_3_/*n*-hexane (2: 3, 11 cm^3^) and more *n*-hexane (11 cm^3^) was added. The solution was briefly sonicated to facilitate crystallization and the compound was isolated by filtration, washed with 20 cm^3^ LP and dried in vacuo to give **5e** (540 mg, 80%). A fraction of this material (95 mg) was further purified by column chromatography (MPLC, 100 g silica, 45 cm^3^ min^−1^ flow rate, LP with a gradient of EtOAc 1–12% within 15 min, then up to 100% EtOAc within 60 min). Yield: 79 mg (corresponds to 67% yield over 2 purification steps); appearance: slightly off-white powder; m.p.: 139–141.5 °C; *R*_*f*_ = 0.48 (hexane/EtOAc, 2:1); ^1^H NMR (DMSO-*d*_*6*_, 200 MHz): *δ* = 3.81 (s, 3H, CH_3_), 7.17–7.48 (m, 9H), 7.58 (t, *J*_3_ = 8.5 Hz, 2H), 7.69 (bs, 1H), 7.99 (s, 1H, H2’), 10.51 (s, 1H, NH) ppm; ^13^C NMR (DMSO-*d*_*6*_, 50 MHz): *δ* = 52.2 (q, CH_3_), 121.3 (d, C2’), 121.8 (d, C2’” & C6’”), 124.0 (d, C4’), 124.2 (d, C6”), 125.1 (d, C6’), 125.8 (d, C4’”), 126.0 (s, C2”), 127.5 (d, C4”*), 128.6 (d, C5”*), 128.7 (d, C5’*), 129.6 (d, C3’” & C5’”), 130.0 (s, C3’), 130.3 (d, C3”), 138.6 (s, C1’), 147.7 (s, C1”), 151.7 (s, C1’”), 166.0 (s, C2*), 166.2 (s, CO_2_*), 171.2 & 171.6 & 171.9 & 172.2 (s, C4 & C6, 2 pairs of rotamers) ppm.

#### Methyl 3-[[4-[4-(1,1-dimethylethyl)phenoxy]-6-phenoxy-1,3,5-triazin-2-yl]amino]benzoate (**5f**, C_27_H_26_N_4_O_4_)

Prepared according to general procedure B using for step 1: 277 mg 2,4,6-trichlorotriazine (1.50 mmol, 1.00 equiv.), 141 mg phenol (1.50 mmol, 1.00 equiv.), 213 mg DIPEA (1.65 mmol, 1.10 equiv.); − 35 °C for 6 h; step 2: 225 mg 4-(1,1-dimethylethyl)phenol (1.50 mmol, 1.00 equiv.), 310 mg DIPEA (2.40 mmol, 1.60 equiv.); 35 °C for 46 h; step 3: 261 mg methyl 3-aminobenzoate (1.73 mmol, 1.15 equiv.), 291 mg DIPEA (2.25 mmol, 1.50 equiv.); 35 °C for 26 h. After the work-up procedure described in general procedure B, the solvent was evaporated completely and the crude material was dissolved in a refluxing mixture of CHCl_3_/*n*-hexane (1:1, 4.5 cm^3^) and more *n*-hexane (8.5 cm^3^) was added. The solution was briefly sonicated to facilitate crystallization and the compound was isolated by filtration, washed with 60 cm^3^ LP, and dried in vacuo to give **5f** (565 mg, 80%). A fraction of this material (235 mg) was further purified by column chromatography (MPLC, 100 g silica, 45 cm^3^ min^−1^ flow rate, LP with a gradient of EtOAc 1 to 12% within 15 min, then up to 100% EtOAc within 60 min). Yield: 194 mg (corresponds to 66% yield over 2 purification steps); appearance: colorless solid; m.p.: 85–86 °C; *R*_*f*_ = 0.53 (hexane/EtOAc, 2:1); ^1^H NMR (DMSO-*d*_*6*_, 200 MHz): *δ* = 1.28 (s, 9H, C(CH_3_)_3_), 3.82 (s, 3H, OCH_3_), 7.14–7.30 (m, 6H), 7.41–7.48 (m, 4H), 7.56 (d, *J*_3_ = 7.7 Hz, 1H), 7.76 (d, *J*_3_ = 7.9 Hz, 1H), 8.04 (s, 1H, H2’), 10.41 (s, 1H, NH) ppm; ^13^C NMR (DMSO-*d*_*6*_, 50 MHz): *δ* = 31.2 (q, C(CH_3_)_3_), 34.2 (s, C(CH_3_)_3_), 52.2 (OCH_3_), 121.2 (d, C2” & C6”), 121.3 (d, C2’), 121.8 (d, C2’” & C6’”), 123.9 (d, C4’), 125.1 (d, C6’), 125.7 (d, C4’”), 126.3 (d, C3” & C5”), 128.7 (d, C5’), 129.6 (d, C3’” & C5’”), 130.0 (s, C3’), 138.9 (s, C1’), 148.0 (s, C4”*), 149.5 (s, C1”*), 151.8 (s, C1’”), 166.0 (s, C2*), 166.2 (s, CO_2_*), 171.7–172.5 (C4 & C6, not resolved) ppm.

#### Methyl 3-[[4-(4-methoxyphenoxy)-6-phenoxy-1,3,5-triazin-2-yl]amino]benzoate (**5g**, C_24_H_20_N_4_O_5_)

Prepared according to general procedure B using for step 1: 277 mg 2,4,6-trichlorotriazine (1.50 mmol, 1.00 equiv.), 141 mg phenol (1.50 mmol, 1.00 equiv.), 213 mg DIPEA (1.65 mmol, 1.10 equiv.); − 35 °C for 7 h; step 2: 186 mg 4-methoxyphenol (1.50 mmol, 1.00 equiv.), 310 mg DIPEA (2.40 mmol, 1.60 equiv.); 35 °C for 47 h; step 3: 261 mg methyl 3-aminobenzoate (1.73 mmol, 1.15 equiv.), 291 mg DIPEA (2.25 mmol, 1.50 equiv.); 35 °C for 23.5 h. After the work-up procedure described in general procedure B, the compound was purified by manual column chromatography (silica, EtOAc/LP) and dried in vacuo. Yield: 569 mg (85%); appearance: colorless solid; m.p.: 56–60 °C; *R*_*f*_ = 0.36 (hexane/EtOAc, 2:1); ^1^H NMR (DMSO-*d*_*6*_, 200 MHz): *δ* = 3.76 (s, 3H, C4”OCH_3_), 3.81 (s, 3H, CO_2_CH_3_), 6.96 (d, *J* = 9.0 Hz, 2H, H3” & H5”), 7.13–7.31 (m, 6H), 7.41–7.48 (m, 2H), 7.56 (d, *J*_3_ = 7.9 Hz, 1H), 7.76 (d, *J* = 7.9 Hz, 1H), 8.04 (s, 1H, H2’), 10.39 (s, 1H, NH) ppm; ^13^C NMR (DMSO-*d*_*6*_, 50 MHz): *δ* = 52.1 (q, CO_2_CH_3_), 55.4 (q, C4”OCH_3_), 114.4 (d, C3” & C5”), 121.1 (d, C2’), 121.7 (d, C2’” & C6’”), 122.6 (d, C2” & C6”), 123.8 (d, C4’), 125.0 (d, C6’), 125.6 (d, C4’”), 128.6 (d, C5’), 129.5 (d, C3’” & C5’”), 129.9 (s, C3’), 138.8 (s, C1’), 145.2 (s, C1”), 151.8 (s, C1’”), 156.8 (s, C4”), 165.9 (s, C2*), 166.1 (s, CO_2_*), 171.7–172.5 (C4 & C6, not resolved) ppm.

#### Methyl 3-[[4-(4-cyanophenoxy)-6-phenoxy-1,3,5-triazin-2-yl]amino]benzoate (**5h**, C_24_H_17_N_5_O_4_)

Prepared according to general procedure B using for step 1: 277 mg 2,4,6-trichlorotriazine (1.50 mmol, 1.00 equiv.), 141 mg phenol (1.50 mmol, 1.00 equiv.), 213 mg DIPEA (1.65 mmol, 1.10 equiv.); − 35 °C for 6 h; step 2: 179 mg 4-hydroxybenzonitrile (1.50 mmol, 1.00 equiv.), 310 mg DIPEA (2.40 mmol, 1.60 equiv.); 35 °C for 46 h; step 3: 261 mg methyl 3-aminobenzoate (1.73 mmol, 1.15 equiv.), 291 mg DIPEA (2.25 mmol, 1.50 equiv.); 35 °C for 26 h. After the work-up procedure described in general procedure B, approx. 10 cm^3^ LP was added to induce precipitation (under sonication) and facilitate evaporation to dryness. The crude material was dissolved in 16 cm^3^ refluxing CHCl_3_ and 16 cm^3^ *n*-hexane was added. After cooling and crystallization were complete, the compound was isolated by filtration, washed with 20 cm^3^ LP, and dried in vacuo to give **5h** (569 mg, 86%). A fraction of this material (223 mg) was further purified by column chromatography (MPLC, 100 g silica, 45 cm^3^ min^−1^ flow rate, LP with a gradient of EtOAc 1 to 12% within 17 min, then up to 100% EtOAc within 60 min). Yield: 183 mg (corresponds to 71% yield over 2 purification steps); appearance: colorless solid; m.p.: 116.5–120 °C; *R*_*f*_ = 0.40 (hexane/EtOAc, 2:1); ^1^H NMR (DMSO-*d*_*6*_, 200 MHz): *δ* = 3.82 (s, 3H, CH_3_), 7.24–7.31 (m, 4H), 7.41–7.60 (m, 5H), 7.71 (bs, 1H), 7.94 (d, *J*_3_ = 8.1 Hz, 2H, H3” & H5”), 8.03 (s, 1H, H2’), 10.50 (s, 1H, NH) ppm; ^13^C NMR (DMSO-*d*_*6*_, 50 MHz): *δ* = 52.1 (q, CH_3_), 108.5 (s, C4”), 118.4 (s, CN), 121.2 (d, C2’), 121.7 (d, C2’” & C6’”), 123.2 (d, C2” & C6”), 124.0 (d, C4’), 125.1 (d, C6’), 125.7 (d, C4’”), 128.6 (d, C5’), 129.5 (d, C3’” & C5’”), 129.9 (s, C3’), 134.0 (d, C3” & C5”), 138.5 (s, C1’), 151.7 (s, C1’”), 155.1 (s, C1”), 165.8 (s, C2*), 166.0 (s, CO_2_*), 170.9–172.3 (C4 & C6, not resolved) ppm.

#### Methyl 3-[[4-(3-nitrophenoxy)-6-phenoxy-1,3,5-triazin-2-yl]amino]benzoate (**5i**, C_23_H_17_N_5_O_6_)

Prepared according to general procedure B using for step 1: 277 mg 2,4,6-trichlorotriazine (1.50 mmol, 1.00 equiv.), 141 mg phenol (1.50 mmol, 1.00 equiv.), 213 mg DIPEA (1.65 mmol, 1.10 equiv.); − 35 °C for 6 h; step 2: 209 mg 3-nitrophenol (1.50 mmol, 1.00 equiv.), 310 mg DIPEA (2.40 mmol, 1.60 equiv.); 40 °C for 46 h; step 3: 261 mg methyl 3-aminobenzoate (1.73 mmol, 1.15 equiv.), 291 mg DIPEA (2.25 mmol, 1.50 equiv.); 40 °C for 24 h. After the work-up procedure described in general procedure B, approx. 5 cm^3^ diethyl ether was added to induce precipitation and facilitate evaporation to dryness. The crude material was dissolved in 8 cm^3^ refluxing CHCl_3_ and 8 cm^3^ *n*-hexane was added. The solution was briefly sonicated to facilitate crystallization and the compound was isolated by filtration and washed with 60 cm^3^ LP. The compound had to be re-crystallized a second time by dissolving it in 17 cm^3^ refluxing CHCl_3_ and adding 17 cm^3^ *n*-hexane. After complete crystallization, the material was isolated by filtration, washed with 25 cm^3^ LP and dried in vacuo. Yield: 474 mg (69%); appearance: colorless powder; m.p.: 165–176 °C; *R*_*f*_ = 0.34 (hexane/EtOAc, 2:1); ^1^H NMR (DMSO-*d*_*6*_, 200 MHz): *δ* = 3.80 (s, 3H, CH_3_), 7.21–7.29 (m, 4H), 7.39–7.46 (m, 2H), 7.57 (d, *J*_3_ = 7.5 Hz, 1H), 7.68–7.80 (m, 3H), 8.05 (s, 1H, H2’), 8.14 (d, *J*_3_ = 7.3 Hz, 1H), 8.21 (s, 1H, H2”), 10.51 (s, 1H, NH) ppm; ^13^C NMR (DMSO-*d*_*6*_, 50 MHz): *δ* = 52.1 (q, CH_3_), 117.4 (d, C2”), 120.6 (d, C4”), 121.2 (d, C2’), 121.7 (d, C2’” & C6’”), 123.9 (d, C4’), 125.1 (d, C6’), 125.7 (d, C4’”), 128.6 (d, C6”*), 128.7 (d, C5’*), 129.4 (d, C3’” & C5’”), 129.9 (s, C3’), 130.7 (d, C5”), 138.5 (s, C1’), 148.3 (s, C3”), 151.7 (s, C1’”*), 151.9 (s, C1”*), 165.8 (s, C2*), 166.1 (s, CO_2_*), 171.1–172.3 (C4 & C6, not resolved) ppm.

#### Methyl 3-[[4-(4-formyl-2-methoxyphenoxy)-6-phenoxy-1,3,5-triazin-2-yl]amino]benzoate (**5j**, C_25_H_20_N_4_O_6_)

Prepared according to general procedure B using for step 1: 277 mg 2,4,6-trichlorotriazine (1.50 mmol, 1.00 equiv.), 141 mg phenol (1.50 mmol, 1.00 equiv.), 213 mg DIPEA (1.65 mmol, 1.10 equiv.); − 35 °C for 6 h; step 2: 228 mg vanillin (1.50 mmol, 1.00 equiv.), 310 mg DIPEA (2.40 mmol, 1.60 equiv.); 40 °C for 46 h; step 3: 261 mg methyl 3-aminobenzoate (1.73 mmol, 1.15 equiv.), 291 mg DIPEA (2.25 mmol, 1.50 equiv.); 40 °C for 24 h. After the work-up procedure described in general procedure B, 2 cm^3^ diethyl ether was added, followed by approx 5 cm^3^ LP to induce crystallization and facilitate evaporation to dryness. As re-crystallization from chloroform/*n*-hexane was not successful, the compound was purified via column chromatography (MPLC, 90 g silica, 45 cm^3^ min^−1^ flow rate, CH_2_Cl_2_ with a gradient of EtOAc (1 to 4% within 60 min) and dried in vacuo. Yield: 545 mg (77%); appearance: colorless crystals; m.p.: 79–82 °C; *R*_*f*_ = 0.24 (hexane/EtOAc, 2:1); ^1^H NMR (DMSO-*d*_*6*_, 200 MHz): *δ* = 3.80 (s, 3H, CO_2_CH_3_), 3.85 (s, 3H, C2”OCH_3_), 7.17–7.30 (m, 4H), 7.40–7.70 (m, 7H), 7.99 (s, 1H, H2’), 10.00 (s, 1H, CHO), 10.46 (s, 1H, NH) ppm; ^13^C NMR (DMSO-*d*_*6*_, 50 MHz): *δ* = 52.1 (q, CO_2_CH_3_), 56.0 (q, C2”OCH_3_), 112.1 (d, C3”), 121.2 (d, C2’), 121.7 (d, C2’” & C6’”), 123.5 (d, C5”*), 123.7 (d, C4’*), 123.9 (d, C6”*), 125.0 (d, C6’), 125.7 (d, C4’”), 128.6 (d, C5’), 129.5 (d, C3’” & C5’”), 129.9 (s, C3’), 135.0 (s, C4”), 138.6 (s, C1’), 145.2 (s, C1”*), 151.68 (s, C2”*), 151.76 (s, C1’”*), 165.8 (s, C2*), 166.0 (s, CO_2_*), 171.2–172.4 (C4 & C6, not resolved), 191.9 (d, CHO) ppm.

#### Methyl 3-[[4-(4-formyl-2-methoxyphenoxy)-6-(4-methoxyphenoxy)-1,3,5-triazin-2-yl]amino]benzoate (**5k**, C_26_H_22_N_4_O_7_)

Prepared according to general procedure B using for step 1: 277 mg 2,4,6-trichlorotriazine (1.50 mmol, 1.00 equiv.), 186 mg 4-methoxyphenol (1.50 mmol, 1.00 equiv.), 213 mg DIPEA (1.65 mmol, 1.10 equiv.); − 35 °C for 7 h; step 2: 228 mg vanillin (1.50 mmol, 1.00 equiv.), 310 mg DIPEA (2.40 mmol, 1.60 equiv.); 40 °C for 47 h; step 3: 261 mg methyl 3-aminobenzoate (1.73 mmol, 1.15 equiv.), 291 mg DIPEA (2.25 mmol, 1.50 equiv.); 40 °C for 23.5 h. After the work-up procedure described in general procedure B, the was compound was purified via column chromatography (MPLC, 90 g silica, 30–35 cm^3^ min^−1^ flow rate, CH_2_Cl_2_ with a gradient of EtOAc 1 to 20%). Mixed fractions were also obtained, which were re-submitted to another chromatographic purification (MPLC, 40 g silica, 35 cm^3^ min^−1^ flow rate, CH_2_Cl_2_ with 4% EtOAc) and dried in vacuo. Yield: 674 mg (90%); appearance: colorless solid; m.p.: 79–82 °C; *R*_*f*_ = 0.18 (hexane/EtOAc, 2:1); ^1^H NMR (DMSO-*d*_*6*_, 200 MHz): *δ* = 3.76 (s, 3H, C4’”OCH_3_), 3.80 (s, 3H, CO_2_CH_3_*), 3.84 (s, 3H, C2”OCH_3_*), 6.95 (d, *J*_3_ = 9.0 Hz, 2H, H3’” & H5’”), 7.13–7.26 (m, 3H), 7.45–7.74 (m, 5H), 8.00 (s, 1H, H2’), 10.00 (s, 1H, CHO), 10.44 (s, 1H, NH) ppm; ^13^C NMR (DMSO-*d*_*6*_, 50 MHz): *δ* = 52.1 (q, CO_2_CH_3_), 55.4 (q, C4’”OCH_3_), 56.1 (q, C2”OCH_3_), 112.2 (d, C3”), 114.4 (d, C3’” & C5’”), 121.3 (d, C2’), 122.6 (d, C2’” & C6’”), 123.6 (d, C5”*), 123.8 (d, C4’*), 124.0 (d, C6”*), 125.1 (d, C6’), 128.7 (d, C5’), 130.0 (s, C3’), 135.0 (s, C4”), 138.7 (s, C1’), 145.2 (s, C1”*), 145.3 (s, C1’”*), 151.7 (s, C2”), 156.9 (s, C4’”), 166.0 (s, C2*), 166.2 (s, CO_2_*), 171.5 & 172.3 (bs, C4 & C6), 192.0 (d, CHO) ppm.

#### Methyl 3-[[4-(4-bromo-2-formylphenoxy)-6-(4-methoxyphenoxy)-1,3,5-triazin-2-yl]amino]benzoate (**5l**, C_25_H_19_BrN_4_O_6_)

Prepared according to general procedure B using for step 1: 277 mg 2,4,6-trichlorotriazine (1.50 mmol, 1.00 equiv.), 186 mg 4-methoxyphenol (1.50 mmol, 1.00 equiv.), 213 mg DIPEA (1.65 mmol, 1.10 equiv.); − 35 °C for 6 h; step 2: 302 mg 5-bromo-2-hydroxybenzaldehyde (1.50 mmol, 1.00 equiv.), 310 mg DIPEA (2.40 mmol, 1.60 equiv.); 40 °C for 46 h; step 3: 261 mg methyl 3-aminobenzoate (1.73 mmol, 1.15 equiv.), 291 mg DIPEA (2.25 mmol, 1.50 equiv.); 40 °C for 24 h. After the work-up procedure described in general procedure B, the compound was purified via column chromatography (MPLC, 90 g silica, 35 cm^3^ min^−1^ flow rate, CH_2_Cl_2_ with 1% EtOAc, gradually increasing to 3% over the course of the separation), which gave 263 mg (32%) of still significantly contaminated material. Thus, 46 mg of the material were submitted to another chromatographic purification (MPLC, 105 g silica, 50 cm^3^ min^−1^ flow rate, LP/EtOAc, 4:1) and then dried in vacuo. Yield: 22 mg (corresponds to 15% yield over 2 purification steps); appearance: colorless solid; m.p.: 180 °C (decomp.); *R*_*f*_ = 0.31 (hexane/EtOAc, 2:1); ^1^H NMR (DMSO-*d*_*6*_, 200 MHz): *δ* = 3.76 (s, 3H, OCH_3_), 3.82 (s, 3H, OCH_3_), 6.96 (d, *J*_3_ = 8.9 Hz, 2H), 7.10–7.34 (m, 3H), 7.43 (d, *J*_3_ = 8.5 Hz, 1H), 7.53–7.81 (m, 2H), 7.89–8.10 (m, 3H), 10.00 (s, 1H, CHO), 10.45 (s, 1H, NH) ppm; ^13^C NMR (DMSO-*d*_*6*_, 50 MHz): *δ* = 52.2 (q, CO_2_CH_3_), 55.4 (q, C4’”OCH_3_), 114.4 (d, C3’” & C5’”), 118.9 (s, C4”), 121.3 (d, C2’), 122.6 (d, C2’” & C6’”), 124.1 (d, C4’), 125.1 (d, C6’), 126.2 (d, C6”*), 128.7 (d, C5’), 129.8 (s, C2”), 130.0 (s, C3’), 132.5 (d, C3”*), 138.2 (d, C5”*), 138.6 (s, C1’), 145.2 (s, C1’”), 151.7 (s, C1”), 156.9 (s, C4’”), 165.9 (s, C2*), 166.0 (s, CO_2_*), 171.5–172.5 (C4 & C6, not resolved), 188.4 (d, CHO) ppm.

#### Methyl 3-[[4-(4-cyanophenoxy)-6-(4-methoxyphenoxy)-1,3,5-triazin-2-yl]amino]benzoate (**5m**, C_25_H_19_N_5_O_5_)

Prepared according to general procedure B using for step 1: 277 mg 2,4,6-trichlorotriazine (1.50 mmol, 1.00 equiv.), 186 mg 4-methoxyphenol (1.50 mmol, 1.00 equiv.), 213 mg DIPEA (1.65 mmol, 1.10 equiv.); − 35 °C for 7 h; step 2: 179 mg 4-hydroxybenzonitrile (1.50 mmol, 1.00 equiv.), 310 mg DIPEA (2.40 mmol, 1.60 equiv.); 40 °C for 47 h; step 3: 261 mg methyl 3-aminobenzoate (1.73 mmol, 1.15 equiv.), 291 mg DIPEA (2.25 mmol, 1.50 equiv.); 40 °C for 23.5 h. After the work-up procedure described in general procedure B, the solvent was evaporated completely and the crude material was dissolved in a refluxing mixture of CHCl_3_/cyclohexane (4:1, 7.5 cm^3^) and more cyclohexane (18 cm^3^) was added. After cooling and crystallization were completed, the compound was isolated by filtration, washed with 25 cm^3^ LP, and dried in vacuo. The material had to be purified further by column chromatography (MPLC, silica, short column, neat CH_2_Cl_2_, 20 cm^3^ min^−1^ flow rate, switching to 30 cm^3^ min^−1^ after 1 h, adding 2% EtOAC after 1 h 20 min and switching to 40 cm^3^ min after the appearance of the product peak (1 h 35 min)). The solvent was evaporated; TLC analysis revealed a byproduct still present. Thus, the compound was submitted to a second short column (MPLC, silica, 25 cm^3^ min^−1^ flow rate, CH_2_Cl_2_ with 2% EtOAc, increasing gradually to 10% with the appearance of the product peak), and dried in vacuo. Yield: 617 mg (88%); appearance: colorless powder; m.p.: 132–134.5 °C; *R*_*f*_ = 0.29 (hexane/EtOAc, 2:1); ^1^H NMR (DMSO-*d*_*6*_, 200 MHz): *δ* = 3.76 (s, 3H, C4’”OCH_3_), 3.82 (s, 3H, CO_2_CH_3_), 6.96 (d, *J*_3_ = 9.0 Hz, 2H, H3’” & H5’”), 7.17 (d, *J*_3_ = 9 Hz, 2H), 7.28 (t, *J*_3_ = 7.9 Hz, 1H), 7.50 (d, *J*_3_ = 8.6 Hz, 2H), 7.58 (d, *J*_3_ = 7.8 Hz, 1H), 7.63–7.82 (m, 1H), 7.94 (d, *J*_3_ = 8.3 Hz, 2H, H3” & H5”), 8.05 (s, 1H, H2’), 10.47 (s, 1H, NH) ppm; ^13^C NMR (DMSO-*d*_*6*_, 50 MHz): *δ* = 52.1 (q, CO_2_CH_3_), 55.4 (q, C4’”OCH_3_), 108.6 (s, C4”), 114.5 (d, C3’” & C5’”), 118.5 (s, CN), 121.3 (d, C2’), 122.6 (d, C2’” & C6’”), 123.2 (d, C2” & C6”), 124.1 (d, C4’), 125.2 (d, C6’), 128.7 (d, C5’), 130.0 (s, C3’), 134.1 (d, C3” & C5”), 138.7 (s, C1’), 145.2 (s, C1’”), 155.2 (s, C1”), 156.9 (s, C4’”), 165.9 (s, C2*), 166.1 (s, CO_2_*), 171.2 & 171.6 & 172.1 & 172.5 (s, C4 & C6, 2 pairs of rotamers) ppm.

### Synthesis of 2,4,6-trisubstitued 1,3,5-triazines—general procedure C

The reaction was performed in an 8 cm^3^ glass vial, using a cryo or thermo block. DIPEA (523 mg, 4.05 mmol, 2.70 equiv.) was added to 282 mg phenol (3.00 mmol, 2.00 equiv.), dissolved in 1.0 cm^3^ THF, and this solution was then added dropwise to a solution of 277 mg 2,4,6-trichlorotriazine (1.50 mmol, 1.00 equiv.) in 2.5 cm^3^ THF at − 35 °C while stirring. To ensure complete transfer, another 0.5 cm^3^ of THF was used to flush all phenol into the reaction mixture. Stirring was continued at − 35 °C for 2 h, then at r. t. for 70 h (step 1) until TLC indicated complete conversion or no significant change in reaction composition. Then, 291 mg DIPEA (2.25 mmol, 1.50 equiv.) was added directly to the reaction mixture, followed by nucleophile PhXH (1.73 mmol, 1.15 equiv.). The reaction was continued at r. t. for 24 h (step 2) and checked by TLC. For work-up, 15 cm^3^ CH_2_Cl_2_ was added to the reaction mixture, followed by 15 cm^3^ 1 N HCl_aq_ and the compound was extracted. The layers were separated, the organic phase washed with water (2 × 15 cm^3^) and then concentrated in vacuo. Further work-up and purification procedures are given at the respective examples.

#### 4,6-Diphenoxy-*N*-phenyl-1,3,5-triazin-2-amine (**6**, C_21_H_16_N_4_O_2_)

Prepared according to general procedure C using for step 1: 277 mg 2,4,6-trichlorotriazine (1.50 mmol, 1.00 equiv.), 282 mg phenol (3.00 mmol, 2.00 equiv.), 523 mg DIPEA (4.05 mmol, 2.70 equiv.); − 35 °C for 2 h, then r. t. for 70 h; step 2: 161 mg aniline (157 mm^3^, 1.73 mmol, 1.15 equiv.), 291 mg DIPEA (2.25 mmol, 1.50 equiv.); r. t. for 24 h. After the work-up procedure described in general procedure A, 2 cm^3^ diethyl ether and 5 cm^3^ LP were added to induce precipitation and facilitate evaporation to dryness. The crude material was dissolved in a refluxing mixture of CHCl_3_/*n*-hexane (3:2, 10 cm^3^) and more *n*-hexane (15 cm^3^) was added; after cooling and complete crystallization, the compound [21, 49, 50] was isolated by filtration, washed with 35 cm^3^ LP, and dried in vacuo. Yield: 452 mg (85%); appearance: colorless solid; m.p.: 156–159 °C; *R*_*f*_ = 0.29 (hexane/EtOAc, 5:1); ^1^H NMR (DMSO-*d*_*6*_, 200 MHz): *δ* = 6.97 (t, *J*_3_ = 7.2 Hz, 1H, H4’), 7.13 (t, *J*_3_ = 7.6 Hz, 2H, H3’ & H5’), 7.22–7.37 (m, 6H), 7.39–7.55 (m, 6H), 10.28 (s, 1H, NH) ppm; ^13^C NMR (DMSO-*d*_*6*_, 50 MHz): *δ* = 120.4 (d, C2’ & C6’), 121.9 (d, C2” & C2’” & C6” & C6’”), 123.2 (d, C4’), 125.7 (d, C4” & C4’”), 128.3 (d, C3’ & C5’) 129.6 (d, C3” & C3’” & C5” & C5’”), 138.4 (s, C1’), 151.9 (s, C1” & C1’”), 165.9 (s, C2), 171.8 & 172.3 (s, C4 & C6, rotamers) ppm.

#### 2,4-Diphenoxy-6-(phenylthio)-1,3,5-triazine (**7**, C_21_H_15_N_3_O_2_S)

Prepared according to general procedure C using for step 1: 277 mg 2,4,6-trichlorotriazine (1.50 mmol, 1.00 equiv.), 282 mg phenol (3.00 mmol, 2.00 equiv.), 523 mg DIPEA (4.05 mmol, 2.70 equiv.); − 35 °C for 2 h, then r. t. for 70 h; step 2: 190 mg thiophenol (176 mm^3^, 1.73 mmol, 1.15 equiv.), 291 mg DIPEA (2.25 mmol, 1.50 equiv.); r. t. for 24 h. After the work-up procedure described in general procedure A, the solvent was evaporated completely and the crude material was dissolved in a refluxing mixture of CHCl_3_/*n*-hexane (3:2, 8.5 cm^3^) and more *n*-hexane (10 cm^3^) was added; after cooling and crystallization were completed, the compound was isolated by filtration and dried in vacuo. Yield: 405 mg (72%); appearance: colorless solid; m.p.: 157–161.5 °C; *R*_*f*_ = 0.38 (hexane/EtOAc, 5:1); ^1^H NMR (DMSO-*d*_*6*_, 200 MHz): *δ* = 7.13–7.29 (m, 6H), 7.30–7.46 (m, 7H), 7.46–7.56 (m, 2H) ppm; ^13^C NMR (DMSO-*d*_*6*_, 50 MHz): *δ* = 121.4 (d, C2’ & C2” & C6’ & C6”), 125.9 (d, C4’ & C4”), 126.3 (s, C1’”), 129.2 (d, C3’” & C5’”), 129.5 (d, C3’ & C3” & C5’ & C5”), 129.7 (d, C4’”), 134.8 (d, C2’” & C6’”), 151.3 (s, C1’ & C1”), 170.6 (s, C2 & C4), 184.8 (s, C6) ppm.

### Synthesis of 1,1-dimethylethyl (4,6-disubstituted-1,3,5-triazin-2-yl)aminobenzoates—general procedure D

The reaction was performed in an 8 cm^3^ glass vial, using a cryo or thermoblock. DIPEA (1.10 equiv.) was added to phenolic compound R^1^PhOH (1.00 equiv.), dissolved in 0.4 cm^3^ THF. This solution was cooled to − 35 °C and then added dropwise to a solution of 2,4,6-trichlorotriazine (1.00 equiv.) in 0.7 cm^3^ THF at − 35 °C while stirring. To ensure complete transfer, another 0.4 cm^3^ of THF was used to flush all phenolic compounds into the reaction mixture. Stirring was continued (step 1) until TLC indicated complete conversion or no significant change in reaction composition. Then, DIPEA (1.60 equiv.) was added to a solution of phenol R^2^PhOH (1.00 equiv.) in 0.4 cm^3^ THF, and this mixture added to the reaction. To ensure complete transfer, another 0.4 cm^3^ of THF was used to flush all phenolic compounds into the reaction mixture. Stirring was continued (step 2) and reaction progress was checked by TLC. Then, DIPEA (1.50 equiv.) was added directly to the reaction mixture, followed by 1,1-dimethylethyl 3-aminobenzoate (1.15 equiv.). The reaction was then continued (step 3) and checked by TLC.

*Work-up by general procedure D1* 10 cm^3^ CH_2_Cl_2_ was added to the reaction mixture, followed by 10 cm^3^ water and the compound was extracted. The layers were separated, the aqueous phase was re-extracted with 5 cm^3^ CH_2_Cl_2_, and the combined organic fractions concentrated in vacuo. Further work-up and purification procedures are given at the respective examples.

*Work-up by general procedure D2* the reaction mixture was taken up with 15 cm^3^ EtOAc, washed with 1 N HCl_aq._ (2 × 10 cm^3^), 10 cm^3^ saturated aq. NaHCO_3_ solution, and 10 cm^3^ brine. The organic phase was concentrated in vacuo. Further work-up and purification are as stated below. Further work-up and purification procedures are given at the respective examples.

#### 1,1-Dimethylethyl 3-[(4,6-diphenoxy-1,3,5-triazin-2-yl)amino]benzoate (**8a**, C_26_H_24_N_4_O_4_)

Prepared according to general procedure D using for step 1: 92 mg 2,4,6-trichlorotriazine (0.50 mmol, 1.00 equiv.), 47 mg phenol (0.50 mmol, 1.00 equiv.), 71 mg DIPEA (0.55 mmol, 1.10 equiv.); − 35 °C for 6 h; step 2: 47 mg phenol (0.50 mmol, 1.00 equiv.), 103 mg DIPEA (0.80 mmol, 1.60 equiv.); r. t. for 46 h; step 3: 111 mg 1,1-dimethylethyl 3-aminobenzoate (0.58 mmol, 1.15 equiv.), 97 mg DIPEA (0.75 mmol, 1.50 equiv.); r. t. for 43 h. After the work-up procedure described in general procedure C1, the compound was purified by column chromatography (MPLC, 90 g silica, 45 cm^3^ min^−1^ flow rate, CH_2_Cl_2_/LP, 1: 5 with a gradient of 1–25% EtOAc within 35 min) and dried in vacuo. Yield: 167 mg (73%); appearance: colorless crystals; m.p.: 208–209 °C; *R*_*f*_ = 0.62 (hexane/EtOAc, 2:1); ^1^H NMR (DMSO-*d*_*6*_, 200 MHz): *δ* = 1.51 (s, 9H, (CH_3_)_3_), 7.14–7.34 (m, 7H), 7.39–7.55 (m, 5H), 7.75 (d, *J*_3_ = 8.3 Hz, 1H), 8.02 (s, 1H, H2’), 10.39 (s, 1H, NH) ppm; ^13^C NMR (DMSO-*d*_*6*_, 50 MHz): *δ* = 27.7 (q, C(CH_3_)_3_), 80.6 (s, C(CH_3_)_3_), 121.3 (d, C2’), 121.8 (d, C2” & C2’” & C6” & C6’”), 123.7 (d, C4’), 124.7 (d, C6’), 125.7 (d, C4” & C4’”), 128.4 (d, C5’), 129.6 (d, C3” & C3’” & C5” & C5’”), 131.7 (s, C3’), 138.7 (s, C1’), 151.8 (s, C1”, C1’”), 164.6 (s, CO_2_), 166.2 (s, C2), 171.8 & 172.2 (bs, C4 & C6, rotamers) ppm.

#### 1,1-Dimethylethyl 3-[[4-(4-chlorophenoxy)-6-phenoxy-1,3,5-triazin-2-yl]amino]benzoate (**8b**, C_26_H_23_ClN_4_O_4_)

Prepared according to general procedure D using for step 1: 92 mg 2,4,6-trichlorotriazine (0.50 mmol, 1.00 equiv.), 47 mg phenol (0.50 mmol, 1.00 equiv.), 71 mg DIPEA (0.55 mmol, 1.10 equiv.); − 35 °C for 6 h; step 2; 64 mg 4-chlorophenol (0.50 mmol, 1.00 equiv.), 103 mg DIPEA (0.80 mmol, 1.60 equiv.); r. t. for 46 h; step 3: 111 mg 1,1-dimethylethyl 3-aminobenzoate (0.58 mmol, 1.15 equiv.), 97 mg DIPEA (0.75 mmol, 1.50 equiv.); 40 °C for 26 h. After the work-up procedure described in general procedure C1, the compound was purified by column chromatography (MPLC, 90 g silica, 45 cm^3^ min^−1^ flow rate, CH_2_Cl_2_) and dried in vacuo. Yield: 217 mg (89%); appearance: off-white solid; m.p.: 172.5–174 °C; *R*_*f*_ = 0.61 (hexane/EtOAc, 2:1); ^1^H NMR (DMSO-*d*_*6*_, 200 MHz): *δ* = 1.53 (s, 9H, (CH_3_)_3_), 7.14–7.37 (m, 6H), 7.39–7.58 (m, 5H), 7.73 (d, *J*_3_ = 8.1 Hz, 1H), 8.04 (s, 1H, H2’), 10.42 (s, 1H, NH) ppm; ^13^C NMR (DMSO-*d*_*6*_, 50 MHz): *δ* = 27.8 (q, C(CH_3_)_3_), 80.7 (s, C(CH_3_)_3_), 121.3 (d, C2’), 121.8 (d, C2’” & C6’”), 123.77 (d, C2” & C6”), 123.85 (d, C4’), 124.8 (d, C6’), 125.7 (d, C4’”), 128.4 (d, C5’), 129.4 (d, C3”* & C5”*), 129.6 (d, C3’”* & C5’”*), 129.9 (s, C4”), 131.7 (s, C3’), 138.6 (s, C1’), 150.6 (s, C1”), 151.8 (s, C1’”), 164.6 (s, CO_2_), 166.1 (s, C2), 171.5–172.4 (C4 & C6, not resolved) ppm.

#### 1,1-Dimethylethyl 3-[[4-(3-chlorophenoxy)-6-phenoxy-1,3,5-triazin-2-yl]amino]benzoate (**8c**, C_26_H_23_ClN_4_O_4_)

Prepared according to general procedure D using for step 1: 92 mg 2,4,6-trichlorotriazine (0.50 mmol, 1.00 equiv.), 47 mg phenol (0.50 mmol, 1.00 equiv.), 71 mg DIPEA (0.55 mmol, 1.10 equiv.); − 35 °C for 6 h; step 2: 64 mg 3-chlorophenol (0.50 mmol, 1.00 equiv., purified by *Kugelrohr* distillation), 103 mg DIPEA (0.80 mmol, 1.60 equiv.); r. t. for 46 h; step 3: 111 mg 1,1-dimethylethyl 3-aminobenzoate (0.58 mmol, 1.15 equiv.), 97 mg DIPEA (0.75 mmol, 1.50 equiv.); 40 °C for 26 h. After the work-up procedure described in general procedure C1, the compound was purified by column chromatography (MPLC, 90 g silica, 45 cm^3^ min^−1^ flow rate, LP/CH_2_Cl_2_/EtOAc, 10: 2:1, with an additional 2% Et_3_N) and dried in vacuo. Yield: 186 mg (76%); appearance: colorless solid; m.p.: 187.5–190.5 °C; *R*_*f*_ = 0.64 (hexane/EtOAc, 2:1); ^1^H NMR (DMSO-*d*_*6*_, 200 MHz): *δ* = 1.52 (s, 9H, (CH_3_)_3_), 7.13–7.60 (m, 11H), 7.77 (d, *J*_3_ = 8.0 Hz, 1H), 8.04 (s, 1H, H2’), 10.45 (s, 1H, NH) ppm; ^13^C NMR (DMSO-*d*_*6*_, 50 MHz): *δ* = 27.7 (q, C(CH_3_)_3_), 80.6 (s, C(CH_3_)_3_), 120.7 (d, C6”), 121.3 (d, C2’), 121.7 (d, C2’” & C6’”), 122.3 (d, C2”), 123.8 (d, C4’), 124.7 (d, C6’), 125.6 (d, C4’”), 125.8 (d, C4”), 128.4 (d, C5’), 129.5 (d, C3’” & C5’”), 130.8 (d, C5”), 131.7 (s, C3’), 133.4 (s, C3”), 138.6 (s, C1’), 151.8 (s, C1’”), 152.4 (s, C1”), 164.5 (s, CO_2_*), 166.1 (s, C2), 171.2–172.2 (C4 & C6, not resolved) ppm.

#### 1,1-Dimethylethyl 3-[[4-(2-chlorophenoxy)-6-phenoxy-1,3,5-triazin-2-yl]amino]benzoate (**8d**, C_26_H_23_ClN_4_O_4_)

Prepared according to general procedure D using for step 1: 92 mg 2,4,6-trichlorotriazine (0.50 mmol, 1.00 equiv.), 47 mg phenol (0.50 mmol, 1.00 equiv.), 71 mg DIPEA (0.55 mmol, 1.10 equiv.); − 35 °C for 6 h; step 2: 64 mg 2-chlorophenol (0.50 mmol, 1.00 equiv.), 103 mg DIPEA (0.80 mmol, 1.60 equiv.); r. t. for 46 h; step 3: 111 mg 1,1-dimethylethyl 3-aminobenzoate (0.58 mmol, 1.15 equiv.), 97 mg DIPEA (0.75 mmol, 1.50 equiv.); 40 °C for 26 h. After the work-up procedure described in general procedure C1, the compound was purified by column chromatography (MPLC, 90 g silica, 45 cm^3^ min^−1^ flow rate, LP/CH_2_Cl_2_/EtOAc, 80: 8: 1, with an additional 2% Et_3_N) and dried in vacuo. Yield: 189 mg (77%); appearance: colorless crystals; m.p.: 191–192 °C; *R*_*f*_ = 0.63 (hexane/EtOAc, 2:1); ^1^H NMR (DMSO-*d*_*6*_, 200 MHz): *δ* = 1.52 (s, 9H, (CH_3_)_3_), 7.11–7.56 (m, 10H), 7.56–7.79 (m, 2H), 7.99 (s, 1H, H2’), 10.48 (s, 1H, NH) ppm; ^13^C NMR (DMSO-*d*_*6*_, 50 MHz): *δ* = 27.7 (q, C(CH_3_)_3_), 80.6 (s, C(CH_3_)_3_), 121.3 (d, C2’), 121.7 (d, C2’” & C6’”), 123.8 (d, C4’), 124.2 (d, C6”), 124.7 (d, C6’), 125.7 (d, C4’”), 125.9 (s, C2”), 127.4 (d, C4”*), 128.4 (d, C5”*), 128.5 (d, C5’*), 129.5 (d, C3’” & C5’”), 130.2 (d, C3”), 131.6 (s, C3’), 138.4 (s, C1’), 147.7 (s, C1”), 151.7 (s, C1’”), 164.5 (s, CO_2_), 166.1 (s, C2), 171.2 & 171.6 & 171.8 & 172.2 (C4 & C6, 2 pairs of rotamers) ppm.

#### 1,1-Dimethylethyl 3-[[4-[4-(1,1-dimethylethyl)phenoxy]-6-phenoxy-1,3,5-triazin-2-yl]amino]benzoate (**8e**, C_30_H_32_N_4_O_4_)

Prepared according to general procedure D using for step 1: 92 mg 2,4,6-trichlorotriazine (0.50 mmol, 1.00 equiv.), 47 mg phenol (0.50 mmol, 1.00 equiv.), 71 mg DIPEA (0.55 mmol, 1.10 equiv.); − 35 °C for 7 h; step 2: 75 mg 4-(1,1-dimethylethyl)phenol (0.50 mmol, 1.00 equiv.), 103 mg DIPEA (0.80 mmol, 1.60 equiv.); 40 °C for 47 h; step 3: 111 mg 1,1-dimethylethyl 3-aminobenzoate (0.58 mmol, 1.15 equiv.), 97 mg DIPEA (0.75 mmol, 1.50 equiv.); 40 °C for 43 h. After the work-up procedure described in general procedure C1, the compound was purified by column chromatography (MPLC, 90 g silica, 45 cm^3^ min^−1^ flow rate, LP/CH_2_Cl_2_, 5:1, with a gradient of EtOAc from 1 to 25% in 35 min) and dried in vacuo. Yield: 170 mg (67%); appearance: colorless solid; m.p.: 139–141 °C; *R*_*f*_ = 0.75 (hexane/EtOAc, 2:1); ^1^H NMR (DMSO-*d*_*6*_, 200 MHz): *δ* = 1.31 (s, 9H, C4”C(CH_3_)_3_), 1.52 (s, 9H, CO_2_C(CH_3_)_3_), 7.10–7.35 (m, 6H), 7.37–7.58 (m, 5H), 7.78 (d, *J*_3_ = 7.3 Hz, 1H), 8.03 (s, 1H, H2’), 10.39 (s, 1H, NH) ppm; ^13^C NMR (DMSO-*d*_*6*_, 50 MHz): *δ* = 27.8 (q, CO_2_C(CH_3_)_3_), 31.3 (q, C4”C(CH_3_)_3_), 34.3 (s, C4”C(CH_3_)_3_), 80.7 (s, CO_2_C(CH_3_)_3_), 121.2 (d, C2” & C6”*), 121.3 (d, C2’*), 121.8 (d, C2’” & C6’”), 123.7 (d, C4’), 124.7 (d, C6’), 125.7 (d, C4’”), 126.2 (d, C3” & C5”), 128.4 (d, C5’), 129.6 (d, C3’” & C5’”), 131.6 (s, C3’), 138.7 (s, C1’), 148.0 (s, C4”*), 149.5 (s, C1”*), 151.8 (s, C1’”), 164.6 (s, CO_2_), 166.2 (s, C2), 171.4–172.3 (C4 & C6, not resolved) ppm.

#### 1,1-Dimethylethyl 3-[[4-(4-methoxyphenoxy)-6-phenoxy-1,3,5-triazin-2-yl]amino]benzoate (**8f**, C_27_H_26_N_4_O_5_)

Prepared according to general procedure D using for step 1: 92 mg 2,4,6-trichlorotriazine (0.50 mmol, 1.00 equiv.), 47 mg phenol (0.50 mmol, 1.00 equiv.), 71 mg DIPEA (0.55 mmol, 1.10 equiv.); − 35 °C for 7 h; step 2: 62 mg 4-methoxyphenol (0.50 mmol, 1.00 equiv.), 103 mg DIPEA (0.80 mmol, 1.60 equiv.); 40 °C for 47 h; step 3: 111 mg 1,1-dimethylethyl 3-aminobenzoate (0.58 mmol, 1.15 equiv.), 97 mg DIPEA (0.75 mmol, 1.50 equiv.); 40 °C for 43 h. After the work-up procedure described in general procedure C2, 2 cm^3^ diethyl ether and 3 cm^3^ LP was added and the oily material was sonicated to induce precipitation. After cooling to 0 °C for 2 h, the precipitate was collected, and 10 cm^3^ LP was added to the supernatant to induce crystallization of a second fraction which was collected by centrifugation. The combined fractions were purified by column chromatography (MPLC, silica, 45 cm^3^ min^−1^ flow rate, LP with a gradient of Et_2_O from 15 to 75% in 80 min) and dried in vacuo. Yield: 167 mg (68%); appearance: colorless crystals; m.p.: 68–70 °C; *R*_*f*_ = 0.56 (hexane/EtOAc, 2:1); ^1^H NMR (DMSO-*d*_*6*_, 200 MHz): *δ* = 1.52 (s, 9H, (CH_3_)_3_), 3.77 (s, 3H, OCH_3_), 6.97 (d, *J*_3_ = 9.1 Hz, 2H, H3” & H5”), 7.12–7.34 (m, 6H), 7.38–7.58 (m, 3H), 7.77 (d, *J*_3_ = 7.5 Hz, 1H), 8.04 (s, 1H, H2’), 10.37 (s, 1H, NH) ppm; ^13^C NMR (DMSO-*d*_*6*_, 50 MHz): *δ* = 27.8 (q, C(CH_3_)_3_), 55.5 (q, OCH_3_), 80.7 (s, C(CH_3_)_3_), 114.5 (d, C3” & C5”), 121.2 (d, C2’), 121.8 (d, C2’” & C6’”), 122.6 (d, C2” & C6”), 123.8 (d, C4’), 124.8 (d, C6’), 125.7 (d, C4’”), 128.5 (d, C5’), 129.6 (d, C3’” & C5’”), 131.7 (s, C3’), 138.7 (s, C1’), 145.2 (s, C1”), 151.8 (s, C1’”), 156.9 (s, C4”), 164.6 (s, CO_2_), 166.2 (s, C2), 171.2–172.4 (C4 & C6, not resolved) ppm.

#### 1,1-Dimethylethyl 3-[[4-(4-cyanophenoxy)-6-phenoxy-1,3,5-triazin-2-yl]amino]benzoate (**8g**, C_27_H_23_N_5_O_4_)

Prepared according to general procedure D using for step 1: 92 mg 2,4,6-trichlorotriazine (0.50 mmol, 1.00 equiv.), 47 mg phenol (0.50 mmol, 1.00 equiv.), 71 mg DIPEA (0.55 mmol, 1.10 equiv.); − 35 °C for 7 h; step 2: 60 mg 4-hydroxybenzonitrile (0.50 mmol, 1.00 equiv.), 103 mg DIPEA (0.80 mmol, 1.60 equiv.); 40 °C for 47 h; step 3: 111 mg 1,1-dimethylethyl 3-aminobenzoate (0.58 mmol, 1.15 equiv.), 97 mg DIPEA (0.75 mmol, 1.50 equiv.); 40 °C for 43 h. After the work-up procedure described in general procedure C2, the compound was purified by column chromatography (MPLC, silica, 45 cm^3^ min^−1^ flow rate, liquid appl. with CH_2_Cl_2_, LP with a gradient of EtOAc from 4 to 76% within 80 min) and dried in vacuo. Yield: 81 mg (33%); appearance: white solid; m.p.: 176.5–178.5 °C; *R*_*f*_ = 0.49 (hexane/EtOAc, 2:1); ^1^H NMR (DMSO-*d*_*6*_, 200 MHz): *δ* = 1.53 (s, 9H, (CH_3_)_3_), 7.17–7.34 (m, 4H), 7.38–7.58 (m, 5H), 7.71 (bs, 1H), 7.94 (d, *J*_3_ = 8.5 Hz, 2H, H3” & H5”), 8.04 (s, 1H, H2’), 10.46 (s, 1H, NH) ppm; ^13^C NMR (DMSO-*d*_*6*_, 50 MHz): *δ* = 27.8 (q, C(CH_3_)_3_), 80.7 (s, C(CH_3_)_3_), 108.5 (s, C4”), 118.5 (s, CN), 121.3 (d, C2’), 121.8 (d, C2’” & C6’”), 123.2 (d, C2” & C6”), 123.9 (d, C4’*), 124.1 (d, C6’*), 125.8 (d, C4’”), 128.5 (d, C5’), 129.6 (d, C3’” & C5’”), 131.7 (s, C3’), 134.1 (d, C3” & C5”), 138.5 (s, C1’), 151.7 (s, C1’”), 155.2 (s, C1”), 164.5 (s, CO_2_), 166.2 (s, C2) ppm, C4 & C6 not visible.

#### 1,1-Dimethylethyl 3-[[4-(3-nitrophenoxy)-6-phenoxy-1,3,5-triazin-2-yl]amino]benzoate (**8h**, C_26_H_23_N_5_O_6_)

Prepared according to general procedure D using for step 1: 92 mg 2,4,6-trichlorotriazine (0.50 mmol, 1.00 equiv.), 47 mg phenol (0.50 mmol, 1.00 equiv.), 71 mg DIPEA (0.55 mmol, 1.10 equiv.); − 35 °C for 7 h; step 2: 70 mg 3-nitrophenol (0.50 mmol, 1.00 equiv.), 103 mg DIPEA (0.80 mmol, 1.60 equiv.); 40 °C for 47 h; step 3: 111 mg 1,1-dimethylethyl 3-aminobenzoate (0.58 mmol, 1.15 equiv.), 97 mg DIPEA (0.75 mmol, 1.50 equiv.); 40 °C for 43 h. After the work-up procedure described in general procedure C2, 1 cm^3^ EtOAc and 9 cm^3^ LP were added and the oily material sonicated to induce precipitation. The supernatant was removed by centrifugation and the compound purified by preparative TLC (LP: EtOAc, 3: 1) and dried in vacuo. Yield: 83 mg (33%); appearance: yellowish solid; m.p.: 137–148 °C; *R*_*f*_ = 0.47 (hexane/EtOAc, 2:1); ^1^H NMR (DMSO-*d*_*6*_, 200 MHz): *δ* = 1.51 (s, 9H, (CH_3_)_3_), 7.16–7.32 (m, 4H), 7.35-7.57 (m, 3H), 7.62-7.84 (m, 3H), 8.04 (s, 1H, H2’), 8.15 (dt, *J*_3_ = 7.2 Hz, *J*_4_ = 2.0 Hz, 1H), 8.22 (s, 1H, H2”), 10.48 (s, 1H, NH) ppm; ^13^C NMR (DMSO-*d*_*6*_, 50 MHz): *δ* = 27.7 (q, C(CH_3_)_3_), 80.7 (s, C(CH_3_)_3_), 117.4 (d, C2”), 120.7 (d, C4”), 121.2 (d, C2’), 121.7 (d, C2’” & C6’”), 124.0 (d, C4’), 124.8 (d, C6’), 125.7 (d, C4’”), 128.5 (d, C6”*), 128.8 (d, C5’*), 129.6 (d, C3’” & C5’”), 130.8 (d, C5”), 131.7 (s, C3’), 138.5 (s, C1’), 148.4 (s, C3”), 151.7 (s, C1’”*), 152.0 (s, C1”*), 164.5 (s, CO_2_), 166.1 (s, C2) ppm, C4 & C6 not visible.

#### 1,1-Dimethylethyl 3-[[4-(4-formyl-2-methoxyphenoxy)-6-phenoxy-1,3,5-triazin-2-yl]amino]benzoate (**8i**, C_28_H_26_N_4_O_6_)

Prepared according to general procedure D using for step 1: 92 mg 2,4,6-trichlorotriazine (0.50 mmol, 1.00 equiv.), 47 mg phenol (0.50 mmol, 1.00 equiv.), 71 mg DIPEA (0.55 mmol, 1.10 equiv.); − 35 °C for 7 h; step 2: 76 mg vanillin (0.50 mmol, 1.00 equiv.), 103 mg DIPEA (0.80 mmol, 1.60 equiv.); 40 °C for 47 h; step 3: 111 mg 1,1-dimethylethyl 3-aminobenzoate (0.58 mmol, 1.15 equiv.), 97 mg DIPEA (0.75 mmol, 1.50 equiv.); 40 °C for 43 h. After the work-up procedure described in general procedure C2, the compound was purified by column chromatography (MPLC, 90 g silica, 45 cm^3^ min^−1^ flow rate, LP with a gradient of EtOAc from 1 to 40% within 1 h) and dried in vacuo. Yield: 141 mg (55%); appearance: colorless solid; m.p.: 76–79 °C; *R*_*f*_ = 0.31 (hexane/EtOAc, 2:1); ^1^H NMR (DMSO-*d*_*6*_, 200 MHz): *δ* = 1.51 (s, 9H, (CH_3_)_3_), 3.86 (s, 3H, OCH_3_), 7.11–7.33 (m, 4H), 7.36–7.55 (m, 4H), 7.56–7.78 (m, 3H), 8.00 (s, 1H, H2’), 10.00 (s, 1H, CHO), 10.43 (s, 1H, NH) ppm; ^13^C NMR (DMSO-*d*_*6*_, 50 MHz): *δ* = 27.8 (q, C(CH_3_)_3_), 56.2 (q, OCH_3_), 80.8 (s, C(CH_3_)_3_), 112.3 (d, C3”), 121.3 (d, C2’), 121.8 (d, C2’” & C6’”), 123.6 (d, C5”*), 123.8 (d, C4’*), 123.9 (d, C6”*), 124.7 (d, C6’), 125.8 (d, C4’”), 128.5 (d, C5’), 129.6 (d, C3’” & C5’”), 131.7 (s, C3’), 135.1 (s, C4”), 138.6 (s, C1’), 145.3 (s, C1”*), 151.70 (s, C2”*) 151.74 (s, C1’”*), 164.6 (s, CO_2_), 166.1 (s, C2), 192.2 (d, CHO) ppm, C4 & C6 not visible.

#### 1,1-Dimethylethyl 3-[[4-(4-formyl-2-methoxyphenoxy)-6-(4-methoxyphenoxy)-1,3,5-triazin-2-yl]amino]benzoate (**8j**, C_29_H_28_N_4_O_7_)

Prepared according to general procedure D using for step 1: 92 mg 2,4,6-trichlorotriazine (0.50 mmol, 1.00 equiv.), 62 mg 4-methoxyphenol (0.50 mmol, 1.00 equiv.), 71 mg DIPEA (0.55 mmol, 1.10 equiv.); − 35 °C for 6 h; step 2: 76 mg vanillin (0.50 mmol, 1.00 equiv.), 103 mg DIPEA (0.80 mmol, 1.60 equiv.); 40 °C for 45 h; step 3: 111 mg 1,1-dimethylethyl 3-aminobenzoate (0.58 mmol, 1.15 equiv.), 97 mg DIPEA (0.75 mmol, 1.50 equiv.); 40 °C for 22 h. After the work-up procedure described in general procedure C2, the compound was purified by column chromatography (MPLC, 105 g silica, 50 cm^3^ min^−1^ flow rate, LP with a gradient of EtOAc from 20 to 40% within 30 min, then to 100% within 15 min) and dried in vacuo. Yield: 162 mg (59%); appearance: colorless solid; m.p.: 88–106 °C; *R*_*f*_ = 0.30 (hexane/EtOAc, 2:1); ^1^H NMR (DMSO-*d*_*6*_, 200 MHz): *δ* = 1.52 (s, 9H, (CH_3_)_3_), 3.76 (s, 3H, C4’”OCH_3_), 3.86 (s, 3H, C2”OCH_3_), 6.96 (d, *J*_3_ = 9.0 Hz, 2H, H3’” & H5’”), 7.10–7.31 (m, 3H), 7.44–7.86 (m, 5H), 8.02 (s, 1H, H2’), 10.01 (s, 1H, CHO), 10.42 (s, 1H, NH) ppm; ^13^C NMR (DMSO-*d*_*6*_, 50 MHz): *δ* = 27.7 (q, C(CH_3_)_3_), 55.4 (q, C4’”OCH_3_), 56.1 (q, C2”OCH_3_), 80.7 (s, C(CH_3_)_3_), 112.2 (d, C3”), 114.5 (d, C3’” & C5’”), 121.2 (d, C2’), 122.6 (d, C2’” & C6’”), 123.6 (d, C5”*), 123.8 (d, C4’*), 123.9 (d, C6”*), 124.6 (d, C6’), 128.5 (d, C5’), 131.7 (s, C3’), 135.0 (s, C4”), 138.6 (s, C1’), 145.2 (s, C1”*), 145.3 (s, C1’”*), 151.8 (s, C2”), 156.9 (s, C4’”), 164.6 (s, CO_2_), 166.1 (s, C2), 192.1 (d, CHO) ppm, C4 & C6 not visible.

#### 1,1-Dimethylethyl 3-[[4-(4-cyanophenoxy)-6-(4-methoxyphenoxy)-1,3,5-triazin-2-yl]amino]benzoate (**8k**, C_28_H_25_N_5_O_5_)

Prepared according to general procedure D using for step 1: 92 mg 2,4,6-trichlorotriazine (0.50 mmol, 1.00 equiv.), 62 mg 4-methoxyphenol (0.50 mmol, 1.00 equiv.), 71 mg DIPEA (0.55 mmol, 1.10 equiv.); − 35 °C for 7 h; step 2: 60 mg 4-hydroxybenzonitrile (0.50 mmol, 1.00 equiv.), 103 mg DIPEA (0.80 mmol, 1.60 equiv.); 40 °C for 47 h; step 3: 111 mg 1,1-dimethylethyl 3-aminobenzoate (0.58 mmol, 1.15 equiv.), 97 mg DIPEA (0.75 mmol, 1.50 equiv.); 40 °C for 43 h. After the work-up procedure described in general procedure C2, the compound was purified by column chromatography (MPLC, 90 g silica, 45 cm^3^ min^−1^ flow rate, LP with a gradient of EtOAc from 10 to 40% within 1 h) and dried in vacuo. Yield: 145 mg (57%); appearance: colorless solid; m.p.: 161–164 °C; *R*_*f*_ = 0.43 (hexane/EtOAc, 2:1); ^1^H NMR (DMSO-*d*_*6*_, 200 MHz): *δ* = 1.53 (s, 9H, (CH_3_)_3_), 3.77 (s, 3H, OCH_3_), 6.98 (d, *J*_3_ = 9.0 Hz, 2H, H3’” & H5’”), 7.11–7.32 (m, 3H), 7.46–7.59 (m, 3H), 7.61–7.84 (m, 1H), 7.94 (d, *J*_3_ = 8.4 Hz, 2H, H3” & H5”), 8.05 (s, 1H, H2’), 10.45 (s, 1H, NH) ppm; ^13^C NMR (DMSO-*d*_*6*_, 50 MHz): *δ* = 27.8 (q, C(CH_3_)_3_), 55.6 (q, C4’”OCH_3_), 80.8 (s, C(CH_3_)_3_), 108.5 (s, C4”), 114.5 (d, C3’” & C5’”), 118.4 (s, CN), 121.3 (d, C2’), 122.6 (d, C2’” & C6’”), 123.2 (d, C2” & C6”), 124.0 (d, C4’), 124.9 (d, C6’), 128.5 (d, C5’), 131.7 (s, C3’), 134.1 (d, C3” & C5”), 138.6 (s, C1’), 145.2 (s, C1’”), 155.2 (s, C1”), 156.9 (s, C4’”), 164.6 (s, CO_2_), 166.1 (s, C2) ppm, C4 & C6 not visible.

### Synthesis of (4,6-disubstituted-1,3,5-triazin-2-yl)aminophenyl methyl alcohols—general procedure E

The reaction was performed using an 8 cm^3^ vial, using a mixture of MeOH/liq. N_2_ for cooling. A vial was charged with alkyl aminobenzoate **5a**–**5g** (1.00 equiv.) and sealed, and the atmosphere changed to argon using standard Schlenk technique. Anhydrous CH_2_Cl_2_ (1.0 cm^3^) was added via syringe, stirred at r. t. until the ester was dissolved, and then cooled in a MeOH/liq. N_2_ bath. Subsequently, a solution of diisobutylaluminium hydride (DIBAL-H, 0.86 mmol cm^−3^) in *n*-hexane was added slowly. The reaction mixture was stirred until TLC indicated complete conversion (in some cases, more DIBAL-H solution was added in the course of the reaction). Then, the reaction was quenched by adding 1.0 cm^3^ 1 M HCl_aq._, stirred for 5 min, and then removed from the cooling bath, followed by the addition of 10 cm^3^ H_2_O. After extraction with CH_2_Cl_2_ (10 cm^3^, then 2 × 5 cm^3^), the solvent was removed in vacuo and the compound was purified as stated below.

#### [4-[(4,6-Diphenoxy-1,3,5-triazin-2-yl)amino]phenyl]methanol (**9a**, C_22_H_18_N_4_O_3_)

Prepared according to general procedure E using 32 mg ethyl aminobenzoate **5a** (0.075 mmol, 1.00 equiv.), 0.33 cm^3^ DIBAL-H (0.285 mmol, 3.80 equiv.); − 70 °C for 2.25 h; then more DIBAL-H (0.05 cm^3^, 0.045 mmol, 0.60 equiv.); − 70 °C for 1 h. After work-up as described in general procedure E, the compound was dried in vacuo and did not require further purification. Yield: 29 mg (quant.); appearance: colorless powder; m.p.: 152.5–154 °C; *R*_*f*_ = 0.36 (hexane/EtOAc, 1:1); ^1^H NMR (DMSO-*d*_*6*_, 200 MHz): *δ* = 4.37 (d, *J*_3_ = 5.6 Hz, 2H, CH_2_), 5.07 (t, *J*_3_ = 5.6 Hz, 1H, OH), 7.04 (d, *J*_3_ = 8.5 Hz, 2H, H3’ & H5’), 7.21–7.54 (m, 12H), 10.20 (s, 1H, NH) ppm; ^13^C NMR (DMSO-*d*_*6*_, 50 MHz): *δ* = 62.5 (t, OCH_2_), 120.0 (d, C2’ & C6’), 121.9 (d, C2” & C2’” & C6” & C6’”), 125.7 (d, C4” & C4’”), 126.6 (d, C3’ & C5’), 129.6 (d, C3” & C3’” & C5” & C5’”), 137.0 (s, C4’*), 137.4 (s, C1’*), 151.9 (s, C1” & C1’”), 165.8 (s, C2), 171.7 & 172.4 (s, C4 & C6, rotamers) ppm.

#### [3-[(4,6-Diphenoxy-1,3,5-triazin-2-yl)amino]phenyl]methanol (**9b**, C_22_H_18_N_4_O_3_)

Prepared according to general procedure E using 31 mg methyl aminobenzoate **5b** (0.075 mmol, 1.00 equiv.), 0.33 cm^3^ DIBAL-H (0.285 mmol, 3.80 equiv.); − 70 °C for 1 h. After work-up as described in general procedure E, the compound was dried in vacuo and did not require further purification. Yield: 29 mg (quant.); appearance: colorless powder; m.p.: 49–52 °C; *R*_*f*_ = 0.36 (hexane/EtOAc, 1:1); ^1^H NMR (DMSO-*d*_*6*_, 200 MHz): *δ* = 4.27 (d, *J*_3_ = 5.7 Hz, 2H, OCH_2_), 5.09 (t, *J*_3_ = 5.7 Hz, 1H, OH), 6.95 (d, *J*_3_ = 7.3 Hz, 1H), 7.07 (t, *J*_3_ = 7.7 Hz, 1H, H5’), 7.20–7.38 (m, 8H), 7.39–7.53 (m, 4H), 10.22 (s, 1H, NH) ppm; ^13^C NMR (DMSO-*d*_*6*_, 50 MHz): *δ* = 62.8 (t, CH_2_), 118.3 (d, C2’*), 118.8 (d, C6’*), 121.3 (d, C4’*), 121.8 (d, C2” & C2’” & C6” & C6’”), 125.6 (d, C4” & C4’”), 127.9 (d, C5’), 129.5 (d, C3” & C3’” & C5” & C5’”), 138.0 (s, C1’), 142.9 (s, C3’), 151.9 (s, C1” & C1’”), 166.0 (s, C2) ppm, C4 & C6 not visible.

#### [3-[[4-(4-Chlorophenoxy)-6-phenoxy-1,3,5-triazin-2-yl]amino]phenyl]methanol (**9c**, C_22_H_17_ClN_4_O_3_)

Prepared according to general procedure E using 34 mg methyl aminobenzoate **5c** (0.075 mmol, 1.00 equiv.), 0.35 cm^3^ DIBAL-H (0.30 mmol, 4.00 equiv.); − 70 °C for 2.25 h; then more DIBAL-H (0.05 cm^3^, 0.045 mmol, 0.60 equiv.); − 70 °C for 1 h. After work-up as described in general procedure E, the compound was re-crystallized from 1.5 cm^3^ CHCl_3_ and 10 cm^3^ *n*-hexane and dried in vacuo. Yield: 28 mg (89%); appearance: colorless solid; m.p.: 110.5–114 °C; *R*_*f*_ = 0.39 (hexane/EtOAc, 1:1); ^1^H NMR (DMSO-*d*_*6*_, 200 MHz): *δ* = 4.24–4.34 (m 2H, OCH_2_), 5.06–5.16 (m, 1H, OH), 6.97 (d, *J*_3_ = 7.0 Hz, 1H), 7.09 (t, *J*_3_ = 7.6 Hz, 1H, H5’), 7.20–7.39 (m, 7H), 7.39–7.57 (m, 4H), 10.24 (s, 1H, NH) ppm; ^13^C NMR (DMSO-*d*_*6*_, 50 MHz): *δ* = 62.8 (t, CH_2_), 118.4 (d, C2’*), 118.9 (d, C6’*), 121.4 (d, C4’*), 121.8 (d, C2’” & C6’”), 123.9 (d, C2” & C6”), 125.7 (d, C4’”), 128.0 (d, C5’), 129.5 (d, C3’”* & C5’”*), 129.6 (d, C3”* & C5”*), 129.9 (s, C4”), 138.0 (s, C1’), 143.0 (s, C3’), 150.7 (s, C1”), 151.8 (s, C1’”), 165.9 (s, C2) ppm, C4 & C6 not visible.

#### [3-[[4-(3-Chlorophenoxy)-6-phenoxy-1,3,5-triazin-2-yl]amino]phenyl]methanol (**9d**, C_22_H_17_ClN_4_O_3_)

Prepared according to general procedure E using 34 mg methyl aminobenzoate **5d** (0.075 mmol, 1.00 equiv.), 0.45 cm^3^ DIBAL-H (0.385 mmol, 5.10 equiv.); 70 °C for 1.75 h. After work-up as described in general procedure E, the compound was purified by column chromatography (MPLC, 8.5 g silica, 8 cm^3^ min^−1^ flow rate, LP/EtOAc 1:1) and dried in vacuo. Yield: 25 mg (78%); appearance: colorless oil; *R*_*f*_ = 0.49 (hexane/EtOAc, 1:1); ^1^H NMR (DMSO-*d*_*6*_, 200 MHz): *δ* = 4.30 (d, *J*_3_ = 4.6 Hz, 2H, OCH_2_), 5.11 (t, *J*_3_ = 5.4 Hz, 1H, OH), 6.97 (d, *J*_3_ = 7.5 Hz, 1H), 7.10 (t, *J*_3_ = 7.7 Hz, 1H, H5’), 7.20–7.41 (m, 7H), 7.41–7.55 (m, 4H), 10.27 (s, 1H, NH) ppm; ^13^C NMR (DMSO-*d*_*6*_, 50 MHz): *δ* = 62.7 (t, CH_2_), 118.4 (d, C2’*), 118.9 (d, C6’*), 120.8 (d, C6”), 121.4 (d, C4’*), 121.7 (d, C2’” & C6’”), 122.3 (d, C2”), 125.6 (d, C4’”*), 125.8 (d, C4”*), 128.0 (d, C5’), 129.5 (d, C3’” & C5’”), 130.9 (d, C5”), 133.3 (s, C3”), 138.0 (s, C1’), 142.9 (s, C3’), 151.8 (s, C1’”), 152.5 (s, C1”), 165.9 (s, C2) ppm, C4 & C6 not visible.

#### [3-[[4-(2-Chlorophenoxy)-6-phenoxy-1,3,5-triazin-2-yl]amino]phenyl]methanol (**9e**, C_22_H_17_ClN_4_O_3_)

Prepared according to general procedure E using 34 mg methyl aminobenzoate **5e** (0.075 mmol, 1.00 equiv.), 0.35 cm^3^ DIBAL-H (0.30 mmol, 4.00 equiv.); − 70 °C for 2 h; then more DIBAL-H (0.10 cm^3^, 0.085 mmol, 1.15 equiv.); − 70 °C for 1.75 h. After work-up as described in general procedure E, the compound was purified by column chromatography (MPLC, 8.5 g silica, 15 cm^3^ min^−1^ flow rate, LP/EtOAc 3: 5) and dried in vacuo. Yield: 32 mg (quant.); appearance: colorless solid; m.p.: 52–54 °C; *R*_*f*_ = 0.44 (hexane/EtOAc, 1:1); ^1^H NMR (DMSO-*d*_*6*_, 200 MHz): *δ* = 4.27 (d, *J*_3_ = 5.3 Hz, 2H, OCH_2_), 5.09 (t, *J*_3_ = 5.6 Hz, 1H, OH), 6.92–7.12 (m, 2H), 7.17–7.53 (m, 10H), 7.63 (d, *J*_3_ = 7.4 Hz, 1H), 10.31 (s, 1H, NH) ppm; ^13^C NMR (DMSO-*d*_*6*_, 50 MHz): *δ* = 62.8 (t, CH_2_), 118.3 (d, C2’*), 118.8 (d, C6’*), 121.4 (d, C4’*), 121.8 (d, C2’” & C6’”), 124.1 (d, C6”), 125.7 (d, C4’”), 125.9 (s, C2”), 127.4 (d, C4”), 127.9 (d, C5’), 128.5 (d, C5”), 129.5 (d, C3’” & C5’”), 130.2 (d, C3”), 137.9 (s, C1’), 142.9 (s, C3’), 147.8 (s, C1”), 151.7 (s, C1’”), 165.8 (s, C2) ppm, C4 & C6 not visible.

#### [3-[[4-[4-(1,1-Dimethylethyl)phenoxy]-6-phenoxy-1,3,5-triazin-2-yl]amino]phenyl]methanol (**9f**, C_26_H_26_N_4_O_3_)

Prepared according to general procedure E using 35 mg methyl aminobenzoate **5f** (0.075 mmol, 1.00 equiv.), 0.45 cm^3^ DIBAL-H (0.385 mmol, 5.10 equiv.); − 70 °C for 1.5 h. After work-up as described in general procedure E, the compound was dried in vacuo and did not require further purification. Yield: 33 mg (quant.); appearance: colorless solid; m.p.: 47–50 °C; *R*_*f*_ = 0.54 (hexane/EtOAc, 1:1); ^1^H NMR (DMSO-*d*_*6*_, 200 MHz): *δ* = 1.31 (s, 9H, (CH_3_)_3_), 4.28 (d, *J*_3_ = 4.5 Hz, 2H, OCH_2_), 5.11 (bs, 1H, OH), 6.95 (d, *J*_3_ = 7.5 Hz, 1H), 7.06 (t, *J*_3_ = 7.7 Hz, 1H, H5’), 7.12–7.21 (m, 2H), 7.22–7.34 (m, 4H), 7.35–7.52 (m, 5H), 10.20 (s, 1H, NH) ppm; ^13^C NMR (DMSO-*d*_*6*_, 50 MHz): *δ* = 31.2 (q, C(CH_3_)_3_), 34.2 (s, C(CH_3_)_3_), 62.8 (t, CH_2_), 118.3 (d, C2’*), 118.8 (d, C6’*), 121.1 (d, C2”* & C6”*), 121.3 (d, C4’*), 121.8 (d, C2’” & C6’”), 125.6 (d, C4’”), 126.2 (d, C3” & C5”), 127.9 (d, C5’), 129.5 (d, C3’” & C5’”), 138.1 (s, C1’), 142.9 (s, C3’), 147.9 (s, C4”*), 149.5 (s, C1”*), 151.8 (s, C1’”), 165.9 (s, C2) ppm, C4 & C6 not visible.

#### [4-[[4-(4-Methoxyphenoxy)-6-phenoxy-1,3,5-triazin-2-yl]amino]phenyl]methanol (**9g**, C_23_H_20_N_4_O_4_)

Prepared according to general procedure E using 33 mg methyl aminobenzoate **5** **g** (0.075 mmol, 1.00 equiv.), 0.45 cm^3^ DIBAL-H (0.385 mmol, 5.10 equiv.); − 70 °C for 1.75 h. After work-up as described in general procedure E, the compound was dried in vacuo and did not require further purification. Yield: 31 mg (quant.); appearance: colorless oil; *R*_*f*_ = 0.32 (hexane/EtOAc, 1:1); ^1^H NMR (DMSO-*d*_*6*_, 200 MHz): *δ* = 3.78 (s, 3H, OCH_3_), 4.28 (d, *J*_3_ = 5.7 Hz, 2H, CH_2_), 5.10 (t, *J*_3_ = 5.7 Hz, 1H, OH), 6.91–7.52 (m, 13H), 10.18 (s, 1H, NH) ppm; ^13^C NMR (DMSO-*d*_*6*_, 50 MHz): *δ* = 55.4 (q, OCH_3_), 62.8 (t, CH_2_), 114.4 (d, C3” & C5”), 118.3 (d, C2’*), 118.8 (d, C6’*), 121.2 (d, C4’*), 121.8 (d, C2’” & C6’”), 122.6 (d, C2” & C6”), 125.6 (d, C4’”), 127.9 (d, C5’), 129.5 (d, C3’” & C5’”), 138.1 (s, C1’), 142.9 (s, C3’), 145.2 (s, C1”), 151.8 (s, C1’”), 156.8 (s, C4”), 165.9 (s, C2) ppm, C4 & C6 not visible.
